# RNAi of AGAMOUS genes in sweetgum alters reproductive organ identity and decreases fruit persistence

**DOI:** 10.1002/pld3.225

**Published:** 2020-05-21

**Authors:** Amy L. Klocko, Amy M. Brunner, Cathleen Ma, Elizabeth Etherington, Kori Rosenstiel, Anna Magnuson, Barbara J. Taylor, Jed Cappellazzi, Thomas Lockwood, Nichole Covarrubias, Manzhu Bao, Jeffrey J. Morrell, Steven H. Strauss, Manzhu Bao, Nichole Covarrubias

**Affiliations:** ^1^ Department of Forest Ecosystems and Society Oregon State University Corvallis OR USA; ^2^ Department of Forest Resources and Environmental Conservation Virginia Tech Blacksburg VA USA; ^3^ Department of Integrative Biology Oregon State University Corvallis OR USA; ^4^ Department of Wood Science and Engineering Oregon State University Corvallis OR USA; ^5^ Department of Biology University of Colorado Colorado Springs Colorado Springs CO USA; ^6^ Key Laboratory of Horticultural Plant Biology Ministry of Education College of Horticulture and Forestry Sciences Huazhong Agricultural University Wuhan 430070 Hubei China; ^7^ 3642 Augusta National Drive S. Salem 97302 OR USA

**Keywords:** AGAMOUS, invasiveness, *Liquidambar*, MADS, RNA interference, tomography

## Abstract

Sweetgums (*Liquidambar*), members of the family Altingiaceae (Altingiales), have inflorescences and floral organs that are distinctive in structure compared with other angiosperms in which the roles of floral homeotic genes have been studied. To begin to understand the role of AGAMOUS (AG)—a floral homeotic gene that has a major role in stamen and carpel development—in development of the monosexual flowers of sweetgum, we used RNAi to reduce the expression of two members of the *AG* subfamily. Because *AG* suppression should induce floral sterility, RNAi might also provide a tool to mitigate the risks of invasiveness—and to reduce the production of its nuisance fruits or allergenic pollen—when sweetgum is used as an exotic shade or forest tree. We tested 33 independent transgenic events and non‐transgenic controls during 10 years in the field. The RNAi‐*AG* sweetgum trees maintained normal growth, phenology, and vivid fall coloration during the 10 years of study, but 8 insertion events had highly modified inflorescence and floral morphology. The modified flowers had anthers and carpels that were converted to flat leaf‐like structures lacking pollen grains and ovules, respectively. The female inflorescences developed into dry papery structures that failed to produce seeds. These infructescences were smaller than control infructescences, and lost a greater percentage of biomass in a controlled decay assay. RNAi against *AG* genes was highly effective at impairing fertility and modifying reproductive development without significant vegetative effects in sweetgum and gave phenotypes distinct from, but similar to, that of *AG* loss of function in other angiosperms.

## 1 INTRODUCTION

Sweetgum (*Liquidambar styraciflua*) inflorescences and flowers are highly distinctive compared with other plant species for which the function of floral development genes has been studied. Sweetgum is a member of the Altingiaceae, a family of wind‐pollinated trees in the highly diverse Saxifragales (Soltis et al., 2013). Phylogenetic studies support that Saxifragales is sister to the rosids (Group et al., 2016; Zeng et al., 2017) or sister to Vitales and together sister to the rosids (Zhang, Wen, & Zimmer, 2016). The flowers are monosexual without a perianth, and flowers are born on compound inflorescences that usually contain both male and female flowers (Ickert‐Bond, Pigg, & Wen, 2005; Wisniewski & Bogle, 1982). Typically, the primary inflorescence stem bears many sessile to short‐pedunculate male inflorescences on its uppermost portion and one to three basal female inflorescences are born on long peduncles. Secondary and tertiary axes are reduced and inflorescences are spherical heads of flowers fused together at their basal portions. Five to nine stamens form in male flowers around the central whorl where two carpel primordia initiate but cease development. Similarly, stamen development is reduced in female flowers but degenerative stamens occasionally produce pollen (Ickert‐Bond et al., 2005; Wisniewski & Bogle, 1982). The two carpels fuse basally, but not at their distal portions. The elongate styles become sclerified and persist in the woody infructescences, which contain ~37 bilocular fruits. Shortly before anthesis, sterile structures of unresolved homology develop around the carpels and appear as braided nob‐like structures in the infructescence. Fruits dehisce in autumn, but the empty infructescences (known colloquially as "gum balls") usually remain on the tree until late winter to early spring and can persist on the ground in a marginally decomposed state for a couple of years (Levia, 2004).

The *AGAMOUS* (*AG)* gene was the first floral homeotic gene to be characterized (Yanofsky et al., 1990), and subsequent work has shown that it plays a conserved role in reproductive organ differentiation under the ABC/D model of floral development (Irish, 2017). Loss of *AG* leads to conversion of carpels and anthers to petals and sepals, and a loss of floral meristem determinacy (Bowman, Smyth, & Meyerowitz, 1989, 1991; Dreni & Kater, 2014). *AG* is highly conserved across flowering species, and reduction in AG function is associated with homeotic conversion of floral whorls and indeterminacy in many herbaceous and woody species such as snapdragon, ranuculids, camellia, apple, plum, and roses (Davies et al., 1999; Dubois et al., 2010; Galimba et al., 2012; Liu, Zhang, Liu, Li, & Lu, 2013; Sun et al., 2014). A genetic duplication early in the evolution of eudicots, resulted in the euAG and PLENA (PLE) clades of the AG‐lineage and most core eudicots have members of both clades that show various degrees of redundancy, subfunctionalization and neofunctionalization (Dreni & Kater, 2014; Kramer, Jaramillo, & di Stilio, 2004). In several rosids and asterids, members of the PLE lineage have roles in fruit development and seed dispersal (reviewed in Dong & Wang, 2015; Ferrandiz & Fourquin, 2014; Garceau, Batson, & Pan, 2017). In the dry dehiscent fruits of Arabidopsis and tobacco, loss or reduction of *SHATTERPROOF (SHP)* activity prevents lignification in the dehiscence zone resulting in indehiscent fruits (Fourquin & Ferrandiz, 2012; Liljegren et al., 2000).

Because of the unusual structure of sweetgum flowers and infructescences, AG suppression should give insights into the distinctive floral and fruit development in the Altingiaceae. Early characterization of one, known at the time as *LAG,* showed that it is expressed in floral primordia in a pattern consistent with that of *AG* genes in other species (Liu, Huang, Ding, & Tauer, 1999). The other *AG* gene has not been characterized in depth, but has a distinct protein sequence (78% identity, was patented by Rottmann, 1999), and is part of the PLE subfamily, as further discussed below. We designed an RNAi construct to target both sweetgum homologs simultaneously. The resulting RNAi‐*AG* trees were planted in field conditions, and their vegetative growth was monitored across growing seasons prior to, as well as after, the onset of flowering. The vegetative effects from suppression of floral homeotic gene activity have only rarely been studied under physiological conditions relevant to growth in wild and/or cultivated environments (e.g., Klocko, Brunner, et al., 2016; Lu et al., 2019).

Sweetgum and many other angiosperm trees and shrubs are popular for ornamental plantings, with many different foliage, flower, and fruit characteristics available. Many are also grown outside of their native range and have the potential to become invasive (Dehnen‐Schmutz, 2011). Notable examples of invasive ornamentals include Japanese Barberry (*Berberis thunbergii*), Norway Maple (*Acer platanoides*), and Bradford or callery pear (*Pyrus calleryana*) (Swearingen & Bargeron, 2016). Once established, long‐distance spread can occur by movement of fruits and seeds. Surprisingly, sweetgum has rarely been considered invasive outside of its native range in the USA, with California a notable exception. There have been observations in Calflora of putative escapes in nearly 20 counties (Gary Falxa, California Native Plant Society, pers. comm., January 10, 2020; https://www.calflora.org/cgi-bin/species_query.cgi?where-calrecnum=8598).

In addition to unwanted dispersal, many have undesirable fruits when used as street trees or around homes. Sweetgum is native to warm temperate regions of eastern North America and Central America, and is well known for its vivid fall color. However, it is also known for its nuisance woody infructescences which persist for long periods in the environment and makes the trees undesirable for urban planting in many areas (Bruegge et al., 2015).

One solution to the problem of invasiveness and undesirable reproductive structures is to plant sterile, fruitless varieties. Some non‐flowering cultivars of sweetgum are available, such as “Rotundiloba,” a variety recommended for use near paved surfaces (Yamaguchi & Hirano, 2006). New varieties of sterile trees can be obtained through a variety of breeding methods (reviews in Ranney, 2004; Vining, Contreras, Ranik, & Strauss, 2012). These include the creation of wide hybrids, polyploidy, random mutagenesis, and somatic cloning of rare wild mutants. Genetic engineering provides an additional means for inducing sterility that is expected to be more targeted and predictable in its effects. Additionally, genetic engineering can be applied directly to the cultivar of interest with the expectation of little to no modification of other characteristics, whereas most other methods, particularly wide crosses and polyploidization, may bring about significant unintended, collateral modification.

There are a wide number of floral genes known whose GE‐assisted suppression or mutation should give male, female, or complete sterility (Vining et al., 2012). Both meiosis and floral development genes are appealing as their disruption often leads to bisexual sterility in monoecious cultivars, thus preventing spread by both pollen and seed. Currently, effective male sterility has been demonstrated under field conditions for poplar, eucalypt, and pine trees expressing barnase under control of a pollen development gene, and female sterility for poplar trees with RNA interference (RNAi) of the floral development genes *LEAFY* (*LFY*) or *AG* (Elorriaga et al., 2014; Klocko, Brunner, et al., 2016; Lu et al., 2019; Zhang et al., 2012). As sweetgum trees produce both male and female flowers on the same tree, a bisexual approach is desirable so that both pollen (that can self‐ or cross‐fertilize feral trees) and seed escape can be avoided.

Based on knowledge of AG‐like genes in sweetgum at the time the project began, we selected two *AG*‐like genes as targets for obtaining bisexually sterile, and ideally fruitless, sweetgum trees. After flowering was initiated, inflorescence and floral structure, seed production, and viability were studied over several years. We found that suppression of *AG* genes by RNAi gave rise to highly unusual, though generally homologous, floral morphologies compared with AG suppression in other angiosperm species. We also found that AG suppression was also an effective and reliable means for eliminating seed production and altering fruit durability, both without detectable effects on autumn foliage coloration, morphology, or stem growth over several years of observation.

## 2 RESULTS

### 2.1 Tree transformation and survival

**FIGURE 1:**
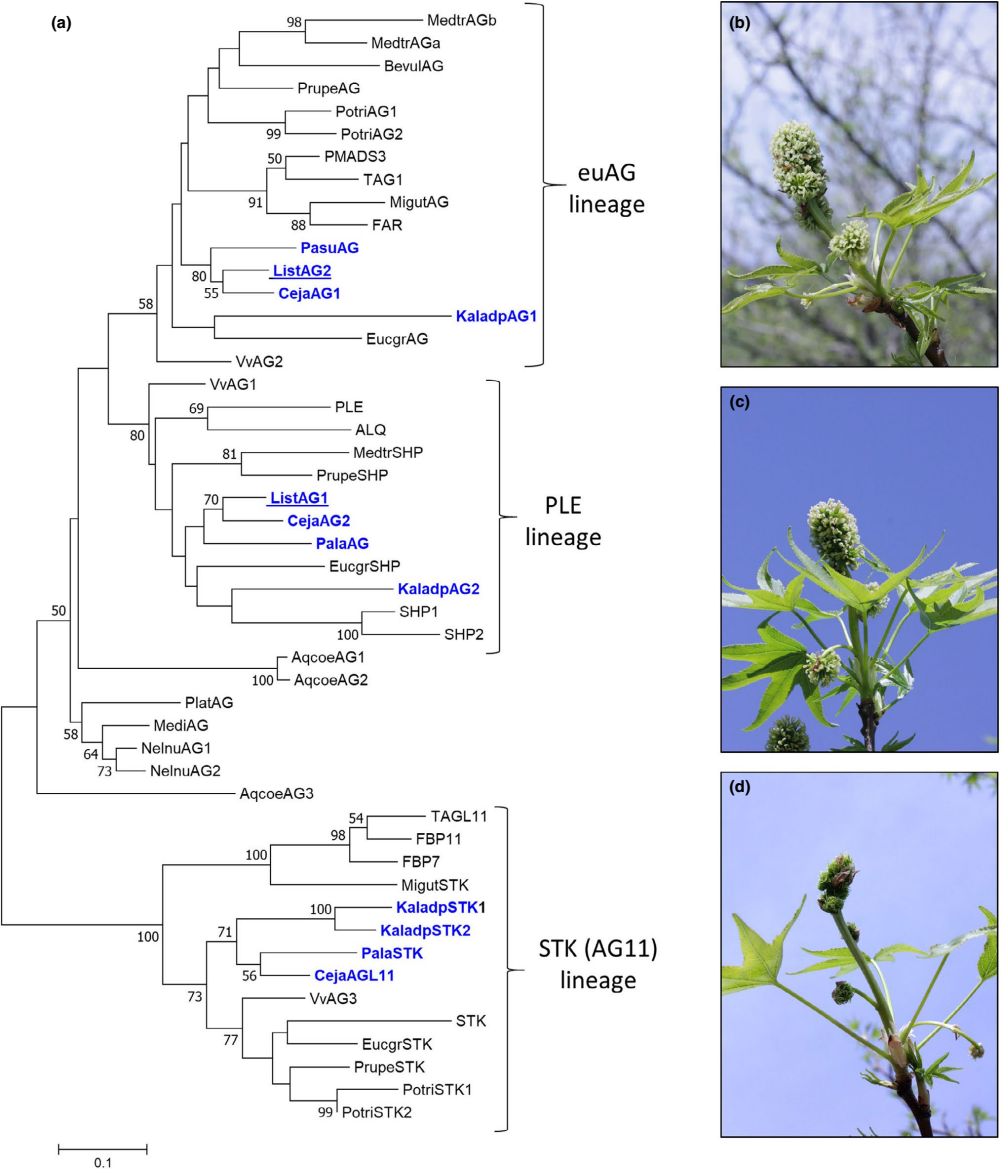
Phylogeny of Eudicot *AG*‐like sequences and alterations in floral morphology of RNAi of both sweetgum *AG* lineage genes. (a) Maximum‐likelihood phylogeny of predicted full‐length peptide sequences of AG‐like sequences from eudicots (species indicators or protein names are in parentheses) including *Aquilegia coerulea* (Aqcoe), *Platanus x hispanica* (Plat), *Meliosma dilleniifolia* (Medi) *Nelumbo nucifera* (Nelnu), *Beta vulgaris* (Bevul), *Petunia x hybrid* (PMADS11,FB7, FB11), *Antirrhinum majus* (FAR, PLE), *Solanum lycopersicum* (ALQ, TAG1, TAG11), *Mimulus guttatus* (Migut), *Vitis vinifera* (Vv), *Paeonia lactiflora* (Pala), *Paeonia suffruticosa* (Pasu), *Kalanchoe fedtschenkoi* (Kaladp), *Eucalyptus grandis* (Eucgr), *Populus trichocarpa* (Potri), *Arabidopsis thaliana* (AG, SHP1, SHP2, STK), *Prunus persica* (Prupe), *Medicago truncatula* (Medtr), *Cercidiphyllum japonicum* (Ceja), and *Liquidambar styraciflua* (List). Saxifragales sequence IDs are shown in blue type, sweetgum *AG*‐like sequence names are underlined. Bootstrap values equal or greater than 50% are shown at nodes. Gene IDs can be found in Table S6. (b) Inflorescence of a wild‐type (WT) control tree opening in field conditions with numerous white stigmas. (c) Some RNAi‐*AG* events had a similar appearance to control inflorescences, (d) other RNAi‐*AG* events had inflorescences that were green and lacked obvious stigmatic tissue and were smaller than WT inflorescences. Inflorescences were imaged in April of 2014

**FIGURE 2:**
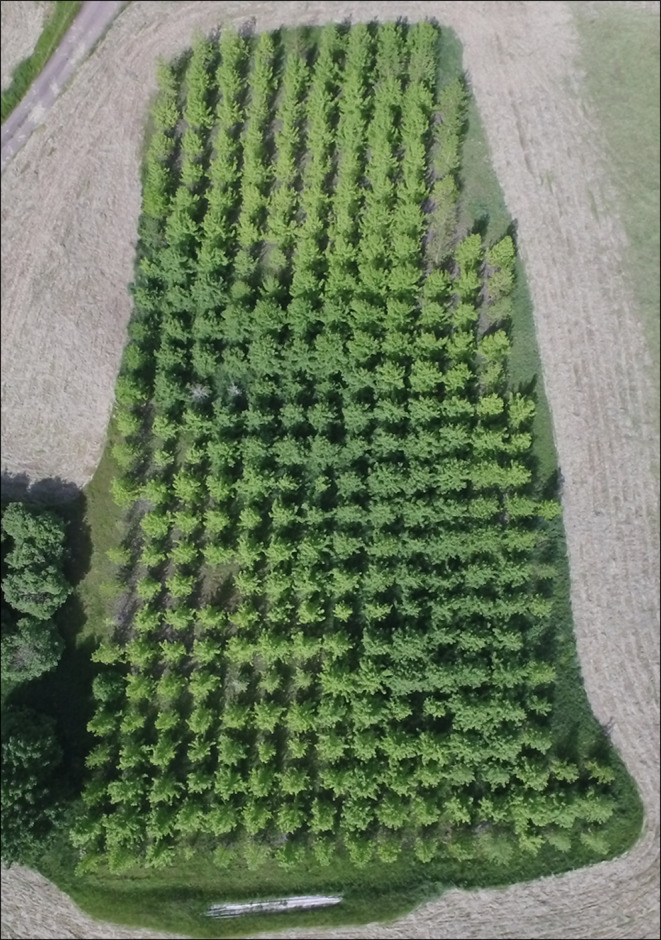
Overhead view of the entire plantation in 2017. Drone view of the entire plantation captured May 2017 shows rows of trees with green spring foliage. A mowed perimeter of grass bounds the plantation, the area was surrounded by a deer exclusion perimeter fence (out of view). A diagonal strip of putatively more fertile soil runs obliquely across the plantation, trees located in this strip have darker green foliage than neighboring trees

Based on data available at the time, we identified two genes from public databases *ListAG1* (hereafter *AG1*) and *ListAG2* (hereafter *AG2)* as being similar to *AG* from *Arabidopsis*. Phylogenetic analysis showed that AG1 and AG2 belong to the PLE and euAG lineages, respectively, and group most closely with other Saxifragales AG family members (Figure 1). We generated an RNAi construct to target both of these *AG*‐like genes and transformed it into the cultivar Worplesdon, a variety known for bright fall foliage (Figure S1). We isolated 33 independent transformation events and planted 4 ramets (clonal replicate trees) per event in two two‐tree row plots (Table S1) along with 12 non‐transgenic control trees. The RNAi‐*AG* trees were established in the field in 2007 as part of a larger trial of different kinds of transgenic trees produced in the Worplesdon cultivar. Tree survival was high; only one ramet from one RNAi‐*AG* event was found to be dead in 2008. All 12 non‐transgenic control and 131 of the 132 RNAi‐*AG* trees were alive in 2017 (Table S1), a survival rate of 99.2%. An aerial view of the plantation captured by an unmanned aerial vehicle (drone) in spring 2017 showed the trees with green spring foliage (Figure 2).

### 2.2 Flowering and floral phenotypes

Trees were screened yearly for floral onset. While it was possible to identify dormant floral buds, destructive sampling was required to differentiate unopened floral buds from unopened vegetative buds. Therefore, trees were scored after inflorescences opened. One non‐transgenic control tree located in the border of the plantation initiated flowering in spring 2012; flowering of interior experimental trees began in 2014. Trees were scored as flowering if they had at least 1 open floral bud; trees from independent gene insertion events (hereafter called “events”) were scored as flowering if at least 1 tree flowered. We found that in 2014 10 of the 131 RNAi‐*AG* ramets flowered (7.6%) representing 7 of the 33 independent events (21.2%). This same year 2 of the 12 non‐transgenic control trees flowered (16.7%, Table S2). In 2015 31 of the 131 RNAi‐*AG* ramets flowered (23.7%) representing 17 of the 33 independent events (51.5%). This same year 4 of the 12 non‐transgenic control trees flowered (33.3%, Table S3).

Floral buds typically initiated opening in April. After flowers fully opened in the field, they were photographed and their phenotypes classified. Control inflorescences had a large upright central floral structure with a rough texture and pinkish‐white coloration. In 2014, four of the RNAi‐*AG* events had inflorescences similar to control trees and were therefore classified as normal (Figure 1). By contrast, three of the RNAi‐*AG* events had inflorescences with a green, nearly moss‐like appearance. We termed these unusual inflorescences “modified,” as they had a very different appearance from the control inflorescences.

**FIGURE 3:**
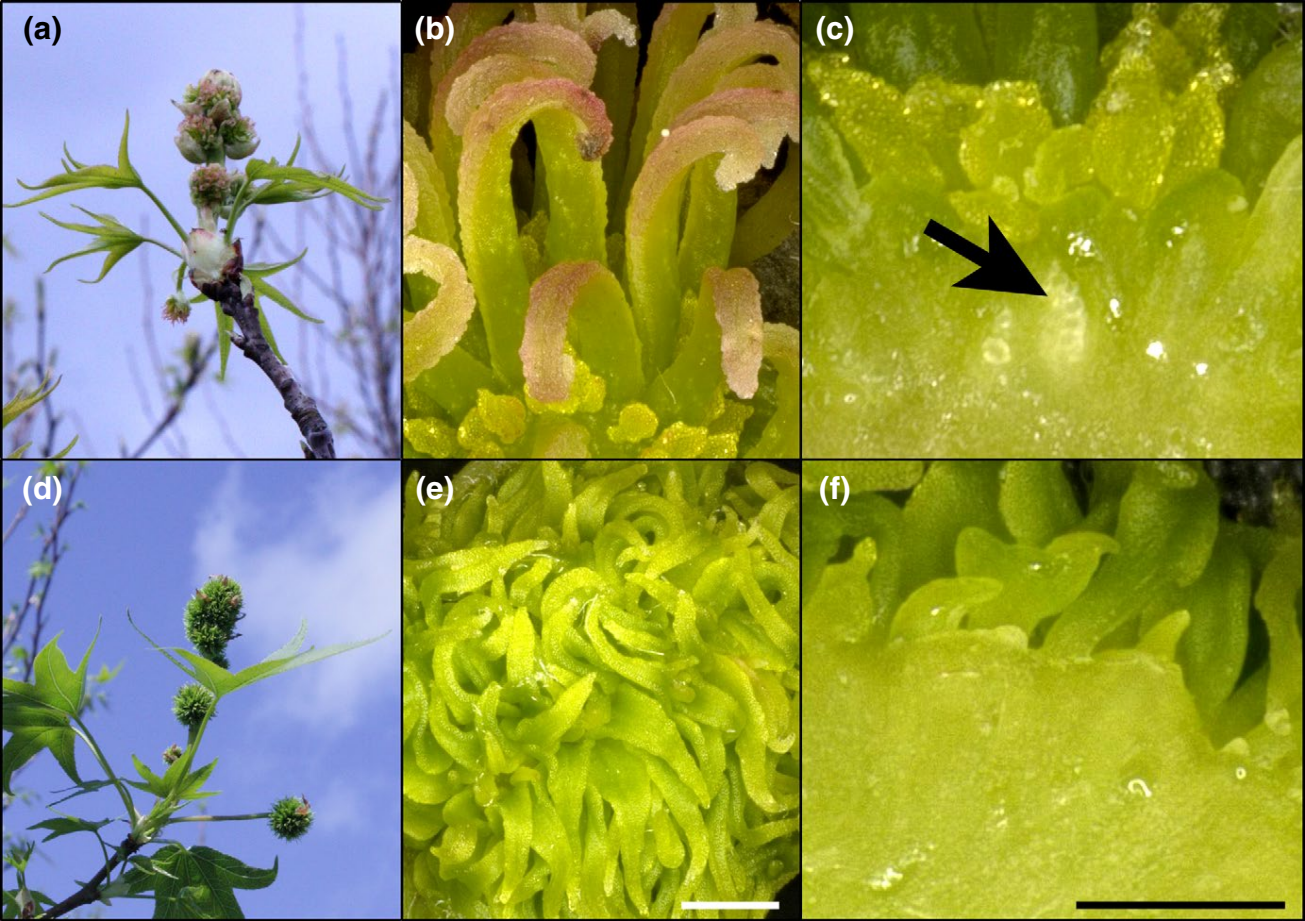
Female RNAi‐*AG* inflorescences lack stigmas or ovules. (a) Female pistilate inflorescences of WT control tree opening in field condition. (b) Microscopic analysis of a WT control female flower exterior showing green styles, pink rough‐textured stigmas, and small yellow undeveloped anthers. (c) A cross‐section through a WT female flower showing ovules (arrow). (d) Female pistilate inflorescences of RNAi‐*AG* tree from event N63‐1opening in field condition. (e) Microscopic analysis of an RNAi‐*AG* event N63‐1 female flower exterior showing flat pointed green floral organs. (f) A cross‐section through an RNAi‐*AG* event N63‐1 female flower showing an absence of ovules. Flowers were collected in April of 2014, scale bars are 1,000 µm

**FIGURE 4:**
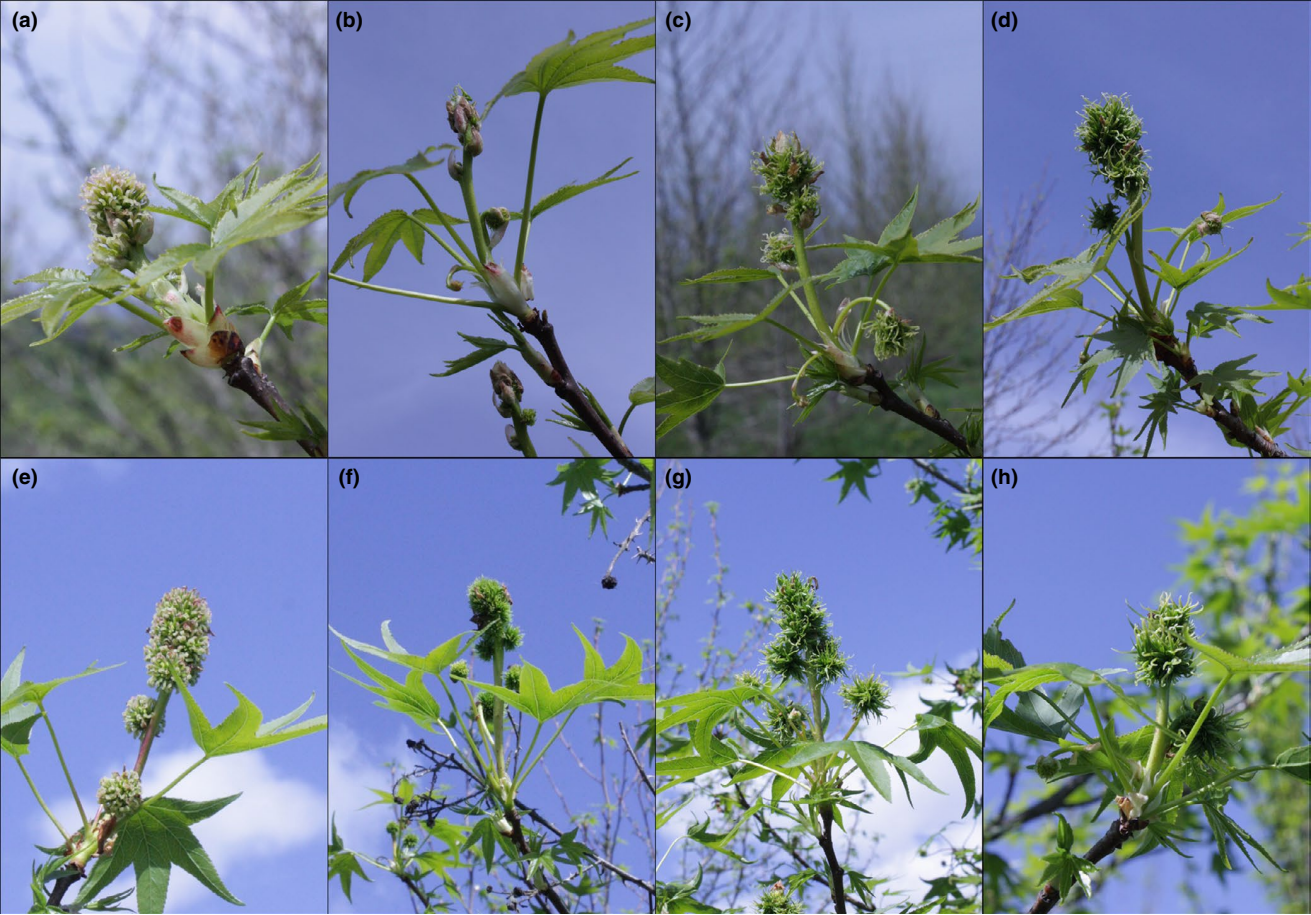
Inflorescence phenotypes were stable across flowering seasons. Female inflorescences of control trees imaged in April of (a) 2014, and (e) 2015, and from three independent RNAi‐*AG* events, N63‐1, P134‐1 and J94‐4, imaged in (b–d) 2014 and (f–h) 2015

**FIGURE 5:**
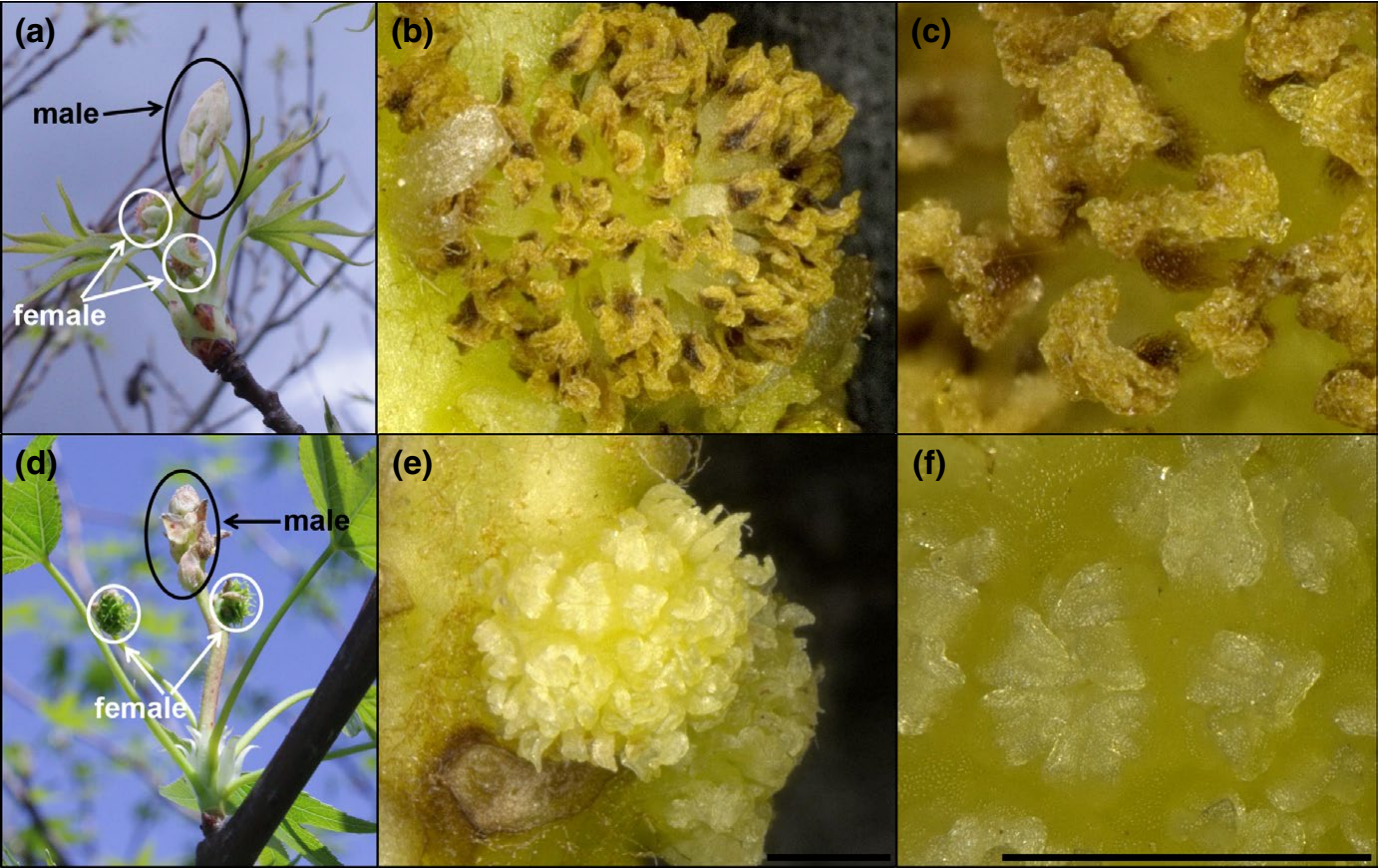
Male RNAi‐AG inflorescences lack well‐formed anthers or pollen grains. (a) Inflorescence of WT control tree opening in field conditions, male staminate flower is encased in bracts; two female pistilate flowers (pink stigmas) are present. (b–c) Microscopic analysis of male WT flowers (bracts removed) showed the presence of numerous rough‐textured stamens. No pollen grains were observed. (d) Inflorescence of RNAi‐*AG* tree event I7‐1 with male flower encased in bracts and two female flowers (green moss‐like balls). (e–f) Microscopic analysis of male RNAi‐*AG* event I7‐1 flowers (bracts removed) showed the presence of flattened triangular organs in the equivalent position of stamens. No pollen grains were observed. Inflorescences were collected in April 2015, scale bars are 1,000 µm

Microscopic analysis of the exterior and interior structures of the control inflorescences collected in April revealed that they had numerous rough‐textured stigmas, tiny undeveloped anthers, and ovaries with numerous ovules (Figure 3). Due to the presence of stigmas and ovules, and the undeveloped nature of the anthers, these inflorescences appeared to be functionally female. The unusual‐appearing RNAi‐*AG* inflorescences had green, pointed structures that lacked stigmatic tissue where styles normally develop and had very tiny green pointed structures in the equivalent positions of the undeveloped control anthers. Sectioning of floral structures to examine their interior form revealed a solid mass of tissue; no ovules were observed in these flowers. We scored floral form again in 2015 and found a total of 8 events had unusual floral phenotypes (Table S4). The floral phenotypes of the 6 events that flowered in both 2014 and 2015 were stable across growing seasons (Figure 4; Table S4). Similar phenotypes were also observed in spring 2017 (Figure S2) and spring 2018. All inflorescences observed in 2014 appeared to be functionally female (Figures 1, 3, 4). In 2015, inflorescences with both female and male inflorescences were identified, allowing us to observe the impacts of *AG* suppression on male floral morphology (Figure 5). The male inflorescences were upright in nature, and above smaller female inflorescences. Male inflorescences had clusters of rough‐textured yellow and brown colored anthers. No pollen grains were observed for these inflorescences in any WT or transgenic events in any of the observation years. Examination of equivalent floral structures in the modified inflorescences revealed the presence of clusters of smooth, pointed, pale yellow organs, and no pollen grains. Other sweetgum trees observed in the same region as the plantation had abundant pollen grains, but these were of a different sweetgum variety than in the experimental plantation.

**FIGURE 6:**
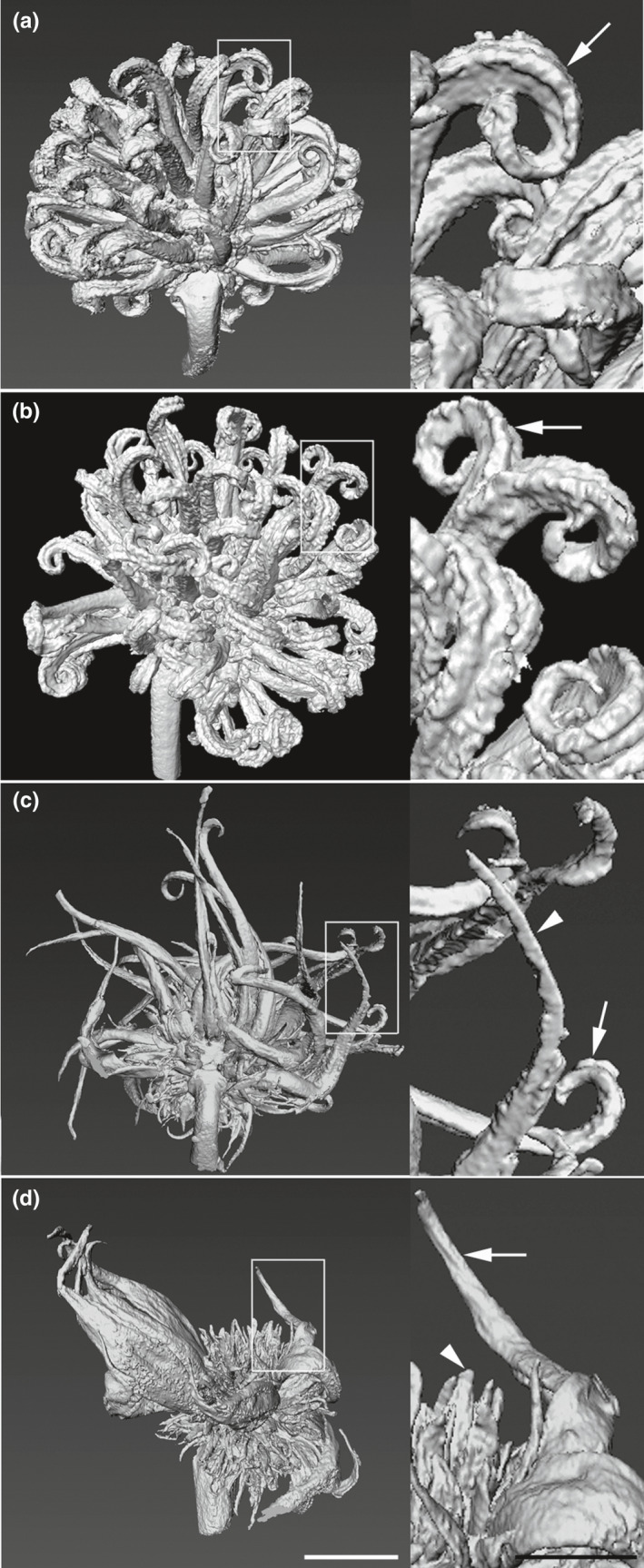
Three‐dimensional surface rendering of wild‐type and altered flowers from micro‐CT scanned images. (a) Wild‐type control female flower (CTR tree J). Higher magnification image (right) shows rough‐textured stigmas (arrow). (b) Wild‐type RNAi‐*AG* female flower event P78‐1. Higher magnification image (right) highlights rough‐textured stigmas (arrow). (c) Modified female RNAi‐*AG* event P134‐1 flower. Higher image (right) shows the diversity of long projections with some (arrowhead) lacking apparent stigmas and others with poorly developed stigmas (arrow). (d) Modified female RNAi‐*AG* event J94‐4 flower bud. Higher image shows a long projection lacking a stigma (arrow) and the presence of flat floral organs (arrowhead). Flowers were collected May of 2018, left scale bar is 1,000 μm, right scale bar is 500 μm

In order to gain more detailed information on the overall structure of the sweetgum inflorescences, we selected young inflorescence from trees representative of control inflorescences, RNAi‐*AG* inflorescences with normal morphology, and female RNAi inflorescences with modified morphology and used these samples for microCT scanning. It was hoped that this method would reveal both the exterior and interior differences of the inflorescences, especially any ovules. We found that this approach led to a detailed view of the exterior surfaces but the interior images yielded insufficient structural contrast (Figure 6). We found that the inflorescence exteriors of control and normal‐flowered RNAi‐AG events were very similar in appearance. Organs were of a regular size, and the stigmatic surfaces had a rough texture. By contract, inflorescences of modified RNAi‐*AG* events had organs that were highly variable in size, were often flat and lacking stigmatic tissue or had very little stigmatic development. Dissection of representative female inflorescences from this same collection also showed the regularity of organ size in control flowers, and the diversity of organ size in the modified inflorescences (Figure S3). The number of organs present in the modified inflorescences was greatly increased, with 158 organs observed in the modified flower, as compared to 105 in the control and 99 in the RNAi event with normal inflorescences. This increase was suggestive of organ duplication, but the complex structure of the inflorescences made it difficult to ascertain the relative positions of the organs.

### 2.3 Gene expression

**FIGURE 7:**
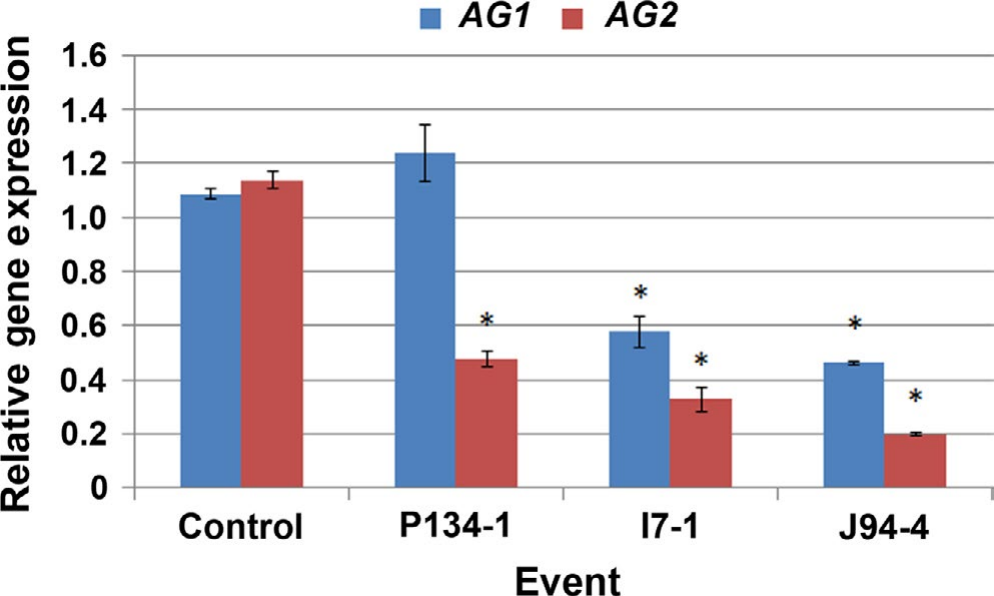
Events with modified inflorescences had reduced expression of both *AG*‐like genes. Relative expression levels of *AG1* and *AG2* in mature floral buds from events with modified inflorescences as compared to control inflorescences. Bars show standard error of the mean of biological replicates, asterisks indicate significant differences in gene expression levels between events and controls (*p *< .05)

We used quantitative real‐time polymerase chain reaction (qRT‐PCR) analysis of gene expression to determine if trees with modified inflorescences had altered expression of the target genes in floral buds. Analysis of *AG1* and *AG2* expression in dormant floral buds showed that all events with modified inflorescences had significantly less expression of *AG2* as compared to control floral buds (Figure 7, mean of 52%). Event I7 and J94 both had significant reduction in *AG1* expression as well (mean of 33.5%).

### 2.4 Seed formation and viability

**FIGURE 8:**
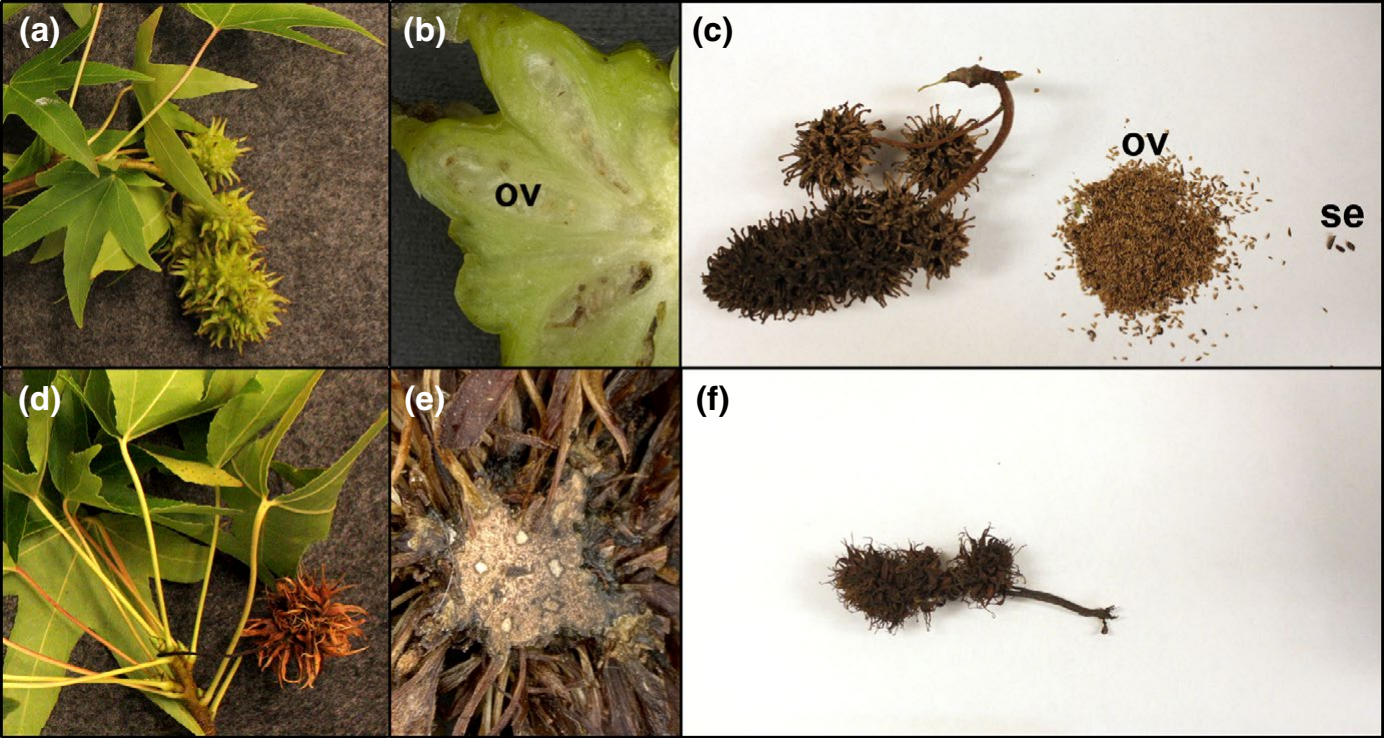
Infructescences from modified RNAi‐*AG* events lacked ovules or seeds. Whole developing Infructescences were collected September 2015 from (a) control and (d) RNAi trees, event N63‐1 is shown. Sectioning of (b) control fruits revealed the presence of many ovules (ov) while sectioning of RNAi fruits (e) showed a solid mass of tissue, event N63‐1 is shown. Collection and vigorous shaking of mature control Infructescences (c) dislodged a large quantity of undeveloped ovules and a small number of seeds (se) while RNAi infructescences (f) yielded no internal contents, event N63‐1 is shown

Starting in 2014, mature infructescences were collected and checked for the presence of seeds (Table S5). While control infructescences contained a larger number of undeveloped ovules and a small number of seeds, infructescences from modified RNAi‐*AG* trees lacked both ovules and seeds (Figure 8). Hand sectioning of these infructescences revealed a small solid core of tissue. For infructescences formed in 2014, no seeds were found for any of the RNAi‐*AG* trees, regardless of floral form; one control tree formed 1 seed, which was viable based on germinability. For infructescences formed in 2015, RNAi‐*AG* trees with normal inflorescences formed 6 seeds, none of which were viable, while RNAi‐*AG* trees with modified inflorescences formed no detectable seeds. Control trees formed 101 seeds, 47 of which were viable. For infructescences formed in 2016, RNAi‐*AG* trees with normal inflorescences formed 13 seeds, two of which were viable, while RNAi‐*AG* trees with modified inflorescences formed no seeds. Control trees formed 159 seeds, 40 of which were viable.

### 2.5 Infructescences characteristics

**FIGURE 9:**
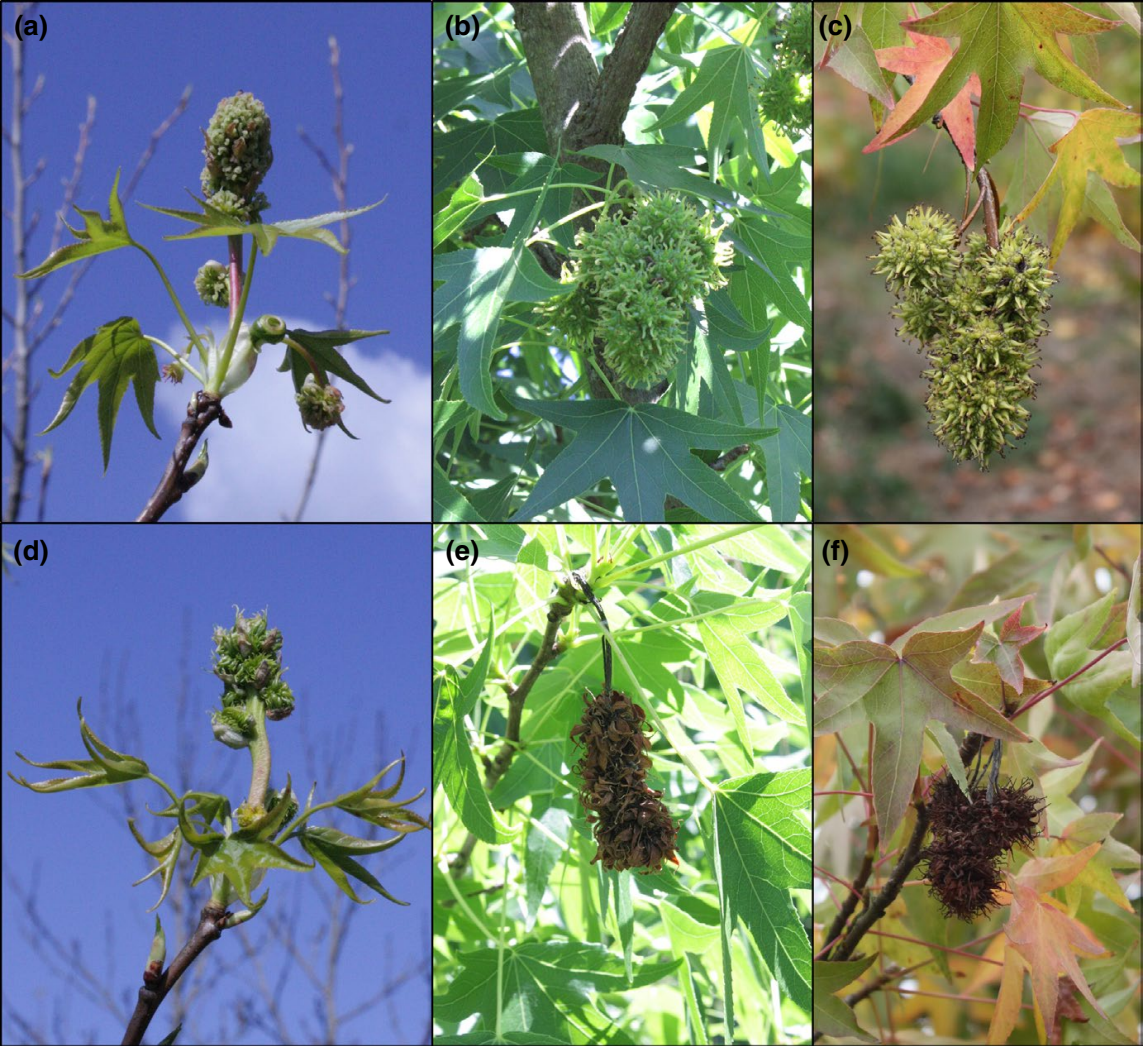
Modified RNAi‐*AG* inflorescences develop into small early‐browning infructescences. (a) Female control inflorescences are fully open in April, (b) enlarge into green infructescences by June, and (c) are still green in October. (d) Modified female RNAi‐*AG* inflorescences are fully open in April (e) are dry and brown by June, and (f) are retained on the tree in October. Example RNAi‐*AG* images are from event I7‐1

**FIGURE 10:**
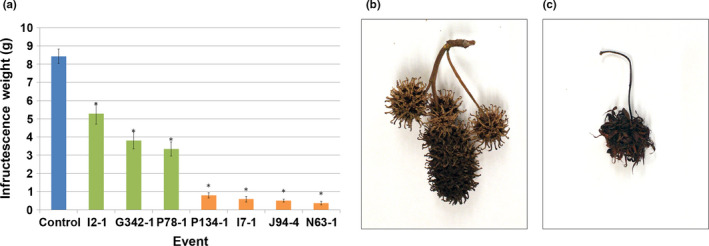
Infructescences from modified RNAi‐*AG* inflorescences were significantly smaller than control infructescences. (a) Quantification of average infructescences weight from control (blue bar), normal (green bars), and modified (orange bars) flower events. Bars show standard error of the mean across fruits, asterisks indicate significant differences as compared to control infructescences (*p *< .05). Examples of (b) control infructescences and (c) RNAi‐*AG* infructescences, event N63‐1 is shown

Infructescences formation was monitored for three years. Control infructescences remained green and continued to enlarge from spring (April) through fall (October) and became fully mature and brown by December (Figure 9). Modified inflorescences showed very little infructescences development, and remained very similar in appearance to the initial bloom. In addition, infructescences were dry and brown in June, much earlier than those from control trees. Like control infructescences, these dry infructescences were retained on the trees and were collected in December for analysis of fruit features. Quantification of mature infructescence size, as determined by dry weight, revealed that infructescences formed from modified inflorescences were significantly smaller (*p* < .001, Figure 10) than control infructescences. These infructescences with a dry, paper‐like appearance, rather than the tough “spikey” infructescences formed on control trees, were also present at harvest in December.

**FIGURE 11:**
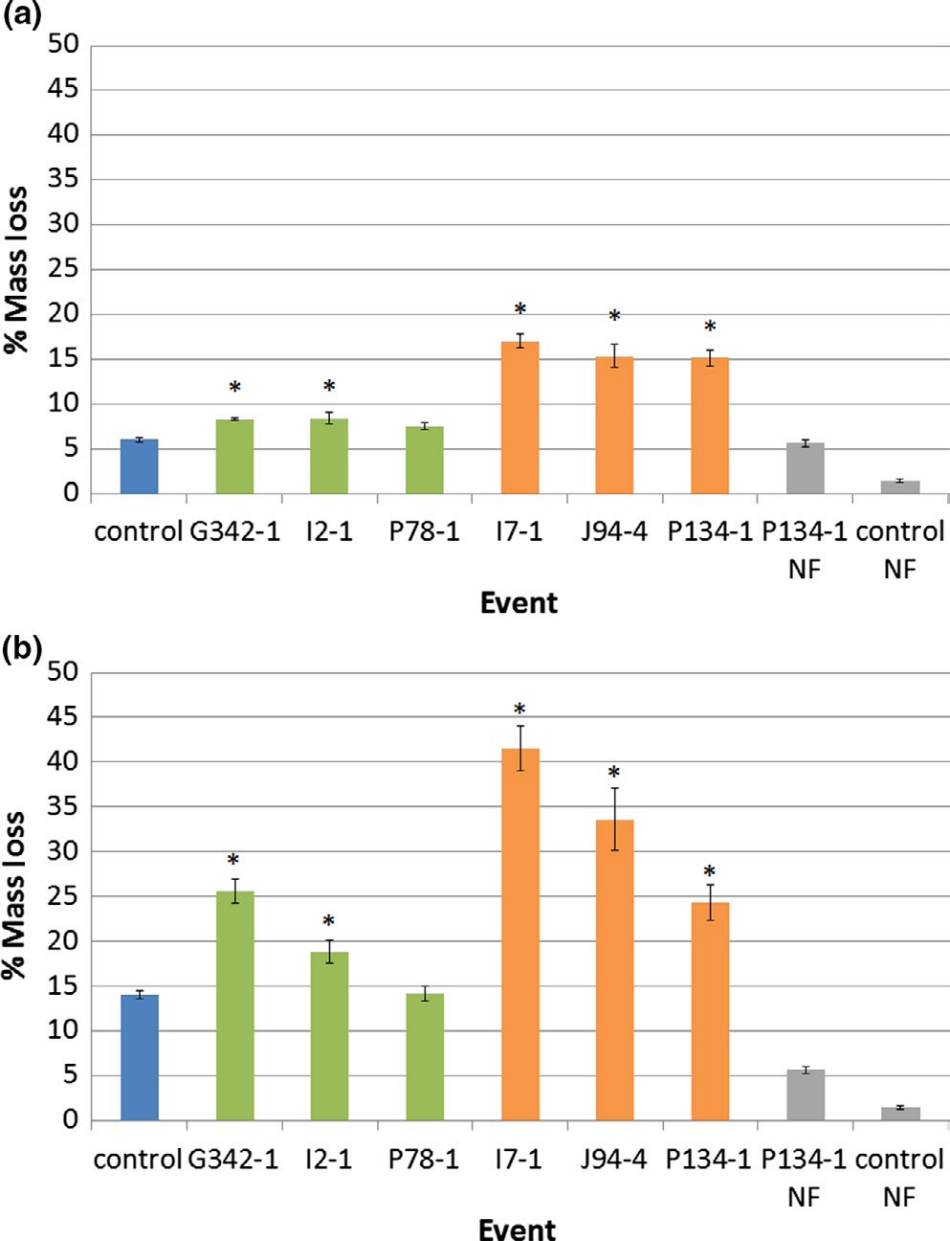
Infructescences from modified inflorescences were less resistant to fungal decomposition than control infructescences. Quantification of total change in dry weight of infructescences treated with (a) brown rot fungus, and (b) white rot fungus. Bars show standard error of the mean, asterisks indicate significant differences as compared to control infructescences (*p* < .05), NF = no fungus controls. Blue bars show non‐transgenic controls, green the RNAi‐AG with normal phenotypes, orange the RNAi‐AG with altered phenotypes, and gray is no fungus (NF) samples of either non‐transgenic control or RNAi with altered phenotypes

We hypothesized that the small papery infructescence might decompose faster than the control infructescence, making them less of a nuisance for street and lawn hygiene. Therefore, we subjected infructescences to a controlled decomposition test to determine infructescence breakdown over time. We tested both a white rot fungus, *Trametes versicolor,* and a brown rot fungus, *Gloeophyllum trabeum* using standard methods (AWPA, 2016). Both of these species of fungi were able to colonize sweetgum infructescences, representative images for white rot colonization are shown, similar results were obtained with brown rot fungi (Figure S4). Infructescence decomposition was calculated as the change in dry weight pre‐ and post‐fungal colonization. Post‐incubation weights included the total biomass of both fungus and infructescences, as the two were inextricably intertwined. Infructescences from modified inflorescences lost a significantly higher percentage of their weight over time than control infructescences, both from the treatment with white rot (33.15%, *p* < .0001) and brown rot fungi (15.85%, *p* < .0001). Examination of individual events showed that infructescences from modified events lost significantly more biomass than control infructescences, and lost more biomass than the two transgenic events with WT‐like inflorescences (Figure 11).

### 2.6 Tree growth and leaf morphology

**FIGURE 12:**
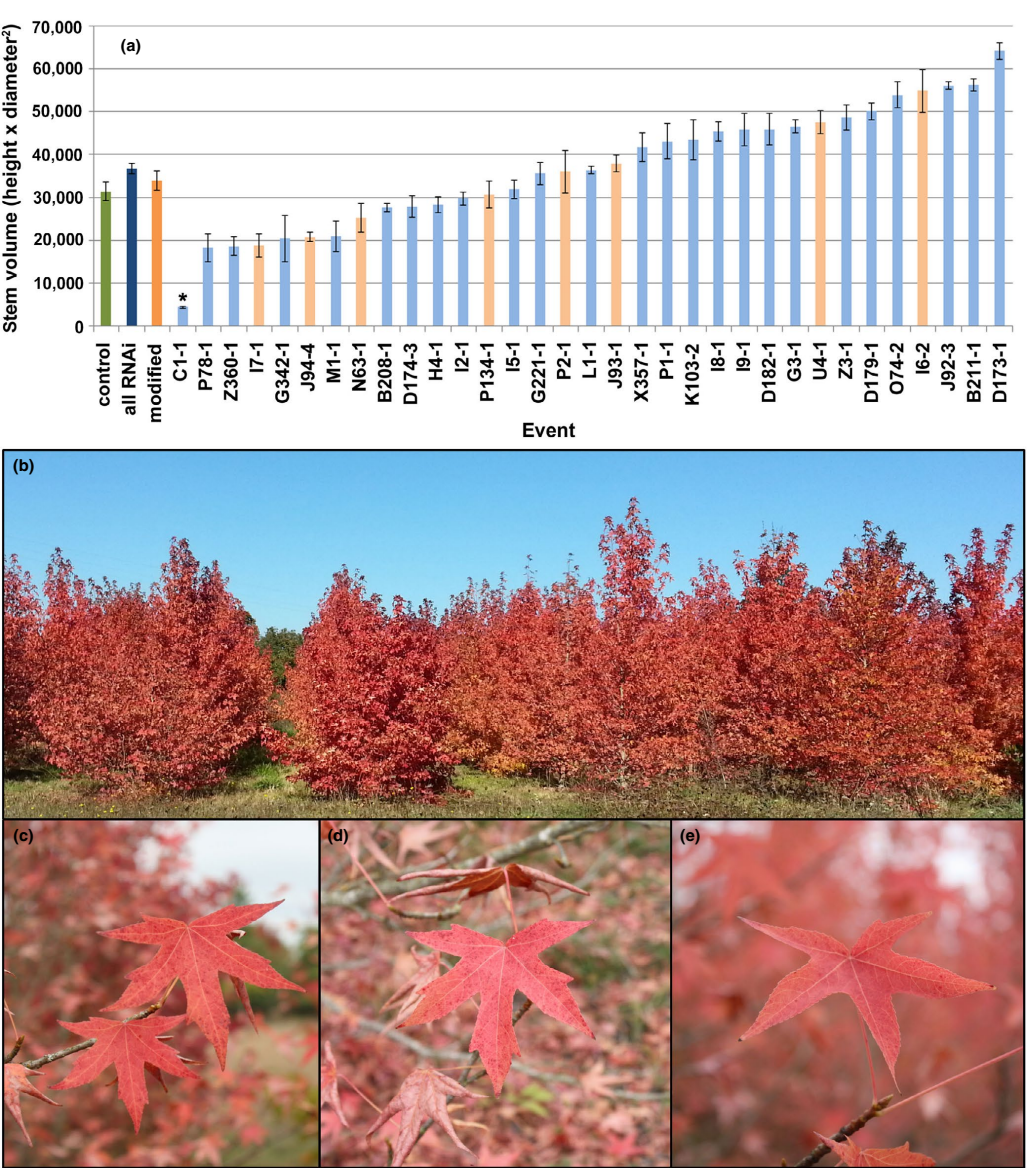
RNAi trees grew as large as or larger than non‐transgenic control trees and had bright fall foliage. (a) Tree size from data collected in 2017 was calculated as stem volume index (height x diameter breast height^2^). Bars show control trees (green bar), all RNAi trees averaged across events (dark blue bar), RNAi trees with modified inflorescences averaged across events (dark orange bar) and individual RNAi events (pale orange and pale blue bars). Events with modified inflorescences are indicated by pale orange bars. Standard error of the mean is shown; asterisks indicate significant differences (*p* < .05). Fall foliage color was scored yearly for the plantation. (b) An overview of the plantation in fall 2013. Leaves imaged in 2015 show typical leaf color of (c) control, (d) normal flowering RNAi event I8‐1, (e) modified flower event J94‐4

Sweetgum trees are grown commercially for wood production and in urban settings as decorative plantings. A significant part of their decorative appeal comes from their fall foliage, which includes a veritable rainbow of cultivars, including deep reds. To determine if RNAi of *AG*‐like genes impacted the vegetative performance or leaf traits of sweetgum trees, we measured tree size and scored fall foliage coloration. We found that tree size, as determined by stem volume index, was not significantly different for RNAi‐*AG* trees after 10 years in the field (*p* = .29) (Figure 12). One RNAi‐*AG* event, C1‐1, showed very poor growth and was, on average, significantly smaller than control trees (*p* = .0026), while six events were significantly larger than control trees (*p *< .05). We did not observe striking alterations in overall tree form between RNAi‐*AG* and non‐transgenic control trees. Worplesdon, the cultivar we selected, has bright reddish fall foliage (Figure 12). Scoring of fall foliage color on a relative intensity scale after one year in the field indicated that RNAi‐*AG* trees were as colorful as control trees (*p* = .28, Figure 12, Figure 5). Similar foliage scoring results were obtained after five growing seasons (*p* = .12).

## 3 DISCUSSION

Studies of AG loss‐of‐function individuals in model species, such as *Arabidopsis*, tobacco, or snapdragon show that reductions in *AG* lead toward conversion of stamens and carpels to petals and sepals (Coen & Meyerowitz, 1991; Kapoor et al., 2002; Yanofsky et al., 1990). Unlike many flowers, sweetgum blossoms lack canonical sepals or petals (Wisniewski & Bogle, 1982). Instead, male inflorescences are clusters of anthers; while female inflorescences are composed of styles with accompanying undeveloped anthers (Figures 1,3, 4, 5, 6). We found that both the anthers and styles in RNAi‐*AG* individuals resembled flat, leaf‐like structures, with a lack of stigmatic tissue (Figures 1,3, 4, 5, 6). Together, these organ identity and meristem traits are classic *AG* loss‐of‐function phenotypes. Therefore, our data support a functional role for the *AG*‐like genes of sweetgum, and add additional evidence for AG function in species with atypical floral form. However, due to the use of a single RNAi construct that targeted both genes, it is difficult to differentiate the functions of the two *AG‐*like genes in sweetgum. We did observe suppression (average of 33% WT levels) of the sweetgum *AG2* gene in floral buds of events with modified inflorescences, and more modest suppression of *AG1* (average of 76% for all events, Figure 7). These expression levels indicate that overall *AG* expression was reduced, but not absent, in our trees, meaning that the phenotypes are likely indicative of partial loss‐of‐function. Thus, it is somewhat surprising that stamens and carpels were almost completely transformed as downregulation of *AG* homologs via RNAi in other plants typically results in partial transformations with organs retaining some characteristics of stamens or carpels (Fourquin & Ferrandiz, 2012; Klocko, Borejsza‐Wysocka, et al., 2016; Lu et al., 2019).

Studies in Arabidopsis indicated that specification of reproductive organ identity and floral determinacy required different levels of AG activity, with determinacy requiring highest AG levels (Mizukami & Ma, 1995). In female inflorescences, the size of the styles, as well as the number present, also varied from control and modified inflorescences, with modified inflorescences showing a greater variety in organ size and increased organ number than controls or RNAi‐AG inflorescences with normal morphology (Figure 6; Figure S3). Such a variation in organ size was also observed in RNAi of *AG* genes in domestic apple, with partial conversion of anthers to petals (Klocko, Borejsza‐Wysocka, et al., 2016). The increase in number could be due to a decrease in floral determinacy, as AG function is known to be important for floral meristem determinacy (reviewed in Sablowski, 2007). However, the closely packed flowers of the inflorescence made it difficult to determine if the increase in organs was on a per flower basis or whether there were more flowers per inflorescence, which would indicate alterations in determinacy of the inflorescence.

A main goal of this work was to assess the impacts of AG reduction on male and female floral fertility. Unfortunately, the impacts on male fertility could not be fully assessed as no pollen grains were observed for any trees in the Worplesdon cultivar, including non‐transgenic controls and Worplesdon trees in the plantation border. This may be because of the tree age; female inflorescences were produced first and had only been ongoing for the three years of observation; functional male flowering may require additional years of maturity. Also, the cultivar we used, Worplesdon, is noted to have “inconspicuous” flowers, perhaps indicative of a reduction in floral form or function (day, 017), and this cultivar may lack robust pollen production. We predict that the structural changes observed in the anthers of RNAi‐*AG* trees (Figure 5) would eliminate or greatly reduce the capacity of these trees to produce pollen grains, as has been observed for RNAi of *AG*‐like genes in domestic apple (Klocko, Borejsza‐Wysocka, et al., 2016) and for suppression of *AG*‐like genes in petunia (Kapoor et al., 2002). In *Arabidopsis*, AG functions to promote pollen grain formation (Ito et al., 2004), making it likely that loss or reduction of sweetgum AG function would impair pollen formation. The structural changes in anthers and loss of seed production in fruits of RNAi‐*AG* trees indicate that both male and female fertility were reduced by suppression of the *AG*‐like genes. It is likely that the evaluation of RNAi events in cultivars which normally produce substantial quantities of pollen would give a better assessment of male fertility.

We observed a loss of not only seed formation (Table S5) in RNAi events with altered inflorescences, but an absence of ovule formation as well (Figures 3, 8). We had hoped to use micro‐CT scanning to obtain better imaging of intact internal structures, with a particular focus on placentas and ovules, but the samples lacked sufficient contrast in density. We also found that RNAi events with externally normal inflorescences had reduced total seed set (0.9 seeds per fruit in 2016) as compared to control trees (14.5 seeds per fruit in 2016, Table S5), perhaps indicative of partial loss‐of‐function phenotypes. The complete lack of ovule formation we observed by dissection and microscopy could be attributed to the homeotic conversion of female floral organs to non‐reproductive structures. However, it might also indicate diversification of function among AG1 and AG2. In tomato, RNAi‐mediated suppression of *TAG1* had almost no effect on stamen and carpel identity, but fruits lacked ovules (Gimenez et al., 2016). RNAi is a classic method for reducing expression of target genes of interest in plants; however, it is possible to suppress non‐target genes that share sequence homology to the intended target (Senthil‐Kumar & Mysore, 2011). Currently, there is a low‐coverage (0.14‐fold depth) sweetgum genome sequence (Staton et al., 2015), making it possible that other *AG*‐like genes have yet to be annotated. Thus, phenotypes might also represent suppression of non‐target genes, such as a member of the *SEEDSTICK* (*STK*) lineage (Figure 1), as was shown to occur in poplar transgenics with RNAi constructs targeting euAG clade members (Lu et al., 2019).

As was reported with RNAi of *AG* in apple, the RNAi‐*AG* sweetgum trees with modified inflorescences still set infructescences (Figures 8, 9). These were significantly smaller than control infructescences (Figure 10, *p* < .001) and with a noticeably different overall form. Importantly, no ovules or seeds were observed in these infructescences, demonstrating an effective loss of female fertility (Table S5; Figure 8). As a primary interest of this work was to test a means to generate fruitless trees, or infructescences with less nuisance potential, we quantified various features. However, the structures present on trees that had modified inflorescences resembled the dried remains of the inflorescences themselves and underwent little change other than turning brown and desiccating months before control inflorescences matured into woody infructescences. For example, the altered infructescences were papery, with a smaller initial mass than controls (Figure 10). Both white and brown rot fungi successfully colonized sweetgum infructescences and decomposed their biomass (Figure S4). Fruits from modified inflorescences lost a greater percentage of their mass than control infructescences (Figure 11), which we interpret as being more decomposed than control infructescences. These findings imply that, under outdoor conditions, the modified infructescences should break down faster to detritus than standard sweetgum infructescences. This trait, and their smaller size, should render the modified infructescences less problematic than wild‐type infructescences, and they may persist for less time.

Our findings demonstrate that *AG*‐like genes are suitable targets for reducing female, and likely, male, fertility in woody species. However, the fact that infructescences were still formed and persisted on the trees from all flowering RNAi‐*AG* events may be due to the incomplete loss of *AG*‐like gene expression (Figure 7). It may be that true nulls, such as those produced by directed mutagenesis methods like CRISPR, would lose all carpel‐like tissue and thus be fruitless. Alternatively, the “infructescences” formed from RNAi events may, in fact, be better described as desiccated inflorescences, as they underwent little, if any, of the expansion observed for control infructescences (Figures 9, 10). If so, then true nulls may retain such dried flower‐like structures, which might actually be vegetative bracts or primordial, undeveloped floral structures.

One advantage of genetic engineering methods is the ability to modify traits in existing cultivars, without the need to breed anew. We did not observe any changes in the quantified vegetative traits of the RNAi‐*AG* trees. Tree size and fall foliage coloration were not significantly affected (Figure 12, Figure 5). While *AG* gene expression can be detected in vegetative tissues of different species including poplar and grape (Brunner et al., 2000; Diaz‐Riquelme, Lijavetzky, Martinez‐Zapater, & Carmona, 2009), reducing *AG*‐like gene expression has not been associated with vegetative impacts. We observed just one event with poor growth (Figure 12); such events are likely somaclonal or epigenetic variants, or a result of insertion site mutagenesis, and could be readily identified and removed from breeding programs. Alternatively, if smaller sized trees are desirable, such events could be retained (semi‐dwarf and sterile).

Genetic engineering methods, including RNAi, show great promise for achieving desired traits in sweetgum and other angiosperm trees. Currently, there are small‐scale or limited commercial plantings of genetically engineered trees, such as virus resistant papaya in Hawaii and other countries (Gonsalves, Lee, & Gonsalves, 2004), insect resistant poplars in China (Hu, Wang, Yan, & Lu, 2014), and growth‐modified eucalypts in Brazil (Canada, 2015). The recent deregulation in the USA of non‐browning Arctic^TM^ apples (Waltz, 2015) and plum pox resistant trees (Scorza et al., 2007) are indicative of increasing interest in use of genetically engineered trees. Many other traits, such as specialty chemical production, disease resistance, and altered wood properties have demonstrated utility in greenhouse and field conditions (reviewed in Chang et al., 2018; Strauss et al., 2017) but have yet to be used beyond research. While the market for sterile transgenic sweetgum is likely of small size, our findings could be applied to many other ornamental species. The current challenge is the need for robust transformation and regeneration systems for woody species, as well as to facilitate social and regulatory acceptance. Recent work in the area of improved transformation of recalcitrant cultivars and species is promising (Lowe et al., 2016; Nagle, Dejardin, Pilate, & Strauss, 2018) and as we have helped to show work in the area of fruitless cultivars may soon, if we may, come to fruition!

## 4 MATERIALS AND METHODS

### 4.1 Plasmid construction and plant transformation

A 605 base pair (bp) fragment corresponding to 305 bp of the *Liquidambar styraciflua* gene *AG2* (GenbankID_AR227777) and 300 bp of the gene *AG1* (GenbankID_AF103903.1) were cloned in sense and antisense orientations in the pHannibal vector (Wesley et al., 2001) generating an inverted repeat flanking an intron. The resulting 35S:*Ls‐AG*:OCSterm was inserted into the binary vector pART27, transformed into *Agrobacterium tumefaciens* strain AGL1, and used for transformation of *Liquidambar styraciflua* cultivar Worplesdon using standard organogenic methods. In brief, explants were co‐cultivated with transformed AGL1 for three days before being transferred to callus and shoot induction medium containing kanamycin and timentin. Shoots were transferred to elongation and rooting medium supplemented with kanamycin and timentin. Shoots that developed roots within two weeks of transfer were subjected to PCR validation of transformation. Verified events were propagated, rooted, and transplanted to small pots. Trees were grown in a greenhouse for 6 months before being transplanted into the field plantation in June 2007. Untransformed *Liquidambar styraciflua* cultivar Worplesdon trees that underwent in vitro propagation, but not organogenesis, were included as non‐transgenic controls.

### 4.2 Phylogenic tree construction

Representative eudicot AG family members were obtained by BLAST P searches of predicted proteome or protein databases (see Table S6 for list of source databases and gene IDs). Full‐length protein sequences were aligned with MUSCLE (Edgar, 2004). A maximum likelihood phylogenetic analysis was performed on the sequence alignment using the JTT + G model, 90% deletion of alignment gaps/missing data and 500 bootstraps for branch support testing with the program MEGA6 (Tamura, Stecher, Peterson, Filipski, & Kumar, 2013).

### 4.3 Field site

Rooted trees were planted as part of a field trial established near to Corvallis, Oregon in June 2007 under an APHIS‐BRS‐approved permit (planting permit 10‐260‐102r‐a1). The 33 events transformed with the RNAi‐AG construct that were the focus of this paper were part of a larger experiment consisting of 1 clone, 3 constructs, 58 transformation events, with 35 to 33 events per construct, and 325 total trees. Four ramets (trees) were planted from each unique transformation event in randomized pairs within a single block. The plantation was bounded by a single‐tree border of 84 non‐experimental trees consisting of a mix of extra ramets from transformation events, non‐transgenic Worplesdon, and non‐transgenic trees of other assorted sweetgum cultivars including Gold Dust^TM^, Rotundiloba, and Moraine. Spacing within rows was 3.05 meters (10 feet); spacing between rows was 3.05 meters (10 feet). Weeds were controlled by hand weeding at tree bases and use of a rotary “weed whacker” between trees. Trees were irrigated with above ground sprinklers from 2007 through 2009. No supplemental water was supplied after this time.

### 4.4 Vegetative phenotype measurements

Tree size (height) was measured every dormant season with a height pole. Tree diameter was measured at 2 inches (50.8 mm) above the ground until they were tall enough for diameter at breast height (DBH) measurements. Tree survival was scored yearly at the time of spring bud flush. Fall leaf color was monitored yearly for all trees on a relative three‐point scale (1 = green, 2 = red coloration appearing, 3 = full red coloration).

### 4.5 Field screening of floral phenotypes and floral bud opening

Trees were surveyed for the presence of inflorescences during the April spring bud flush; no experimental floral buds were observed prior to 2014. Open inflorescences were photographed using a Canon Rebel XSI digital camera. Representative inflorescences were collected and imaged using a Keyence digital microscope VHX‐6000 for detailed analysis of floral morphology.

### 4.6 Micro‐computed tomography (CT) scanning of open flowers

Young open inflorescences were collected May 8, 2018 and selected for analysis by CT scanning using a (Hamamatsu L10711‐19, Hamamatsu Corp.). Samples were wrapped individually wrapped in parafilm and stacked into sample cylinders for scanning. Scan parameters were 70 kVp, 80 μA, with an exposure time of 1.5 s and total scan time of 9 hr. Data sets were reconstructed into 3D volumes from the x‐ray projection data using the Avizo 9.4 program (for details see Supplementary Methods).

### 4.7 Field screening for floral buds and dormant bud collection for gene expression analysis

Trees were examined for the presence of dormant floral buds on February 14, 2017. By this date, the floral buds were larger and plumper than the vegetative buds, and contained developing flowers which were large enough to see by the unaided eye. Branches with dormant buds were collected; bud identity (floral or vegetative) was confirmed by longitudinal sectioning with a clean razor blade. Confirmed floral buds were immediately flash frozen in liquid nitrogen for total RNA extraction and analysis of gene expression.

### 4.8 Expression analysis of *AG1* and *AG2* via qPCR

Total RNA was isolated floral buds collected on February 14, 2017 using a CTAB‐based RNA extraction method (Gambino, Perrone, & Gribaudo, 2008). RNA was treated with DNase (DNase I, Amplification Grade, Invitrogen) according to the manufacturer's protocol. SuperScript III Reverse Transcriptase (Invitrogen) was used for cDNA synthesis following the manufacturer's recommendations. Quantitative RT‐PCR was performed using a StepOnePlus real‐time PCR system (Applied Biosystems) and Platinum SYBR Green qPCR SuperMix with ROX reference dye (Invitrogen). The housekeeping gene *ACTIN* (*ACT*) was used as a reference, and three technical replicates and two biological replicates were used for each reaction. The following gene‐specific primers were used: *AG1* (5'‐TGCCCAGTTTTACCAGCAAG‐3' and 5'‐GCTGATGCCTTTCTCCAACC‐3'), *AG2* (5'‐AGCTTTCAACCACGGCTTTC −3' and 5'‐CCACGGGTAGAGAAGACGAG‐3'), *ACT2* (5'‐CAATCATGAAGTGTGATGTGGA‐3', and 5' GCACGATGTTGCCATAGAGA‐3'). Primers for the *ACT* gene were designed based on sequences deposited from a low‐coverage sequence of the sweetgum genome (Staton et al., 2015). The PCR program consisted of an initial melt of 95°C for 10 min, followed by 40 cycles of 95°C for 15 s and 60°C for 1 min, followed by melt curve analysis with a temperature increase of 0.2°C/s. The relative amount of gene expression was determined using the ΔΔCt method.

### 4.9 Floral dissection and imaging

Female inflorescences were collected on May 8, 2018. Representative inflorescences were selected and imaged on a gray fabric background using an iPad. Inflorescences were hand‐dissected using tweezers and a razor blade to remove styles and stigmas (or equivalently positioned organs) starting with organs nearest the stem and moving upwards. Organs were arranged in a grid with those first removed in the upper left corner, and filled from left to right and top to bottom. The dissected organs were imaged using the iPad.

### 4.10 Infructescence collection and seed viability testing

Mature infructescences were collected yearly in December 2014 and 2015. Infructescences from inflorescences which opened in 2016 did not fully mature into infructescences until January of 2017 and were collected at this time. Mature infructescences were checked for viable seeds using a standard protocol (Sun et al., 2014). In brief, infructescences were air‐dried for one week until valves, if present, opened. Infructescences were then shaken vigorously to dislodge seeds, if any. Infructescences were visually inspected for retained seeds, which were removed with tweezers. Seeds were vernalized in damp filter paper for 4 weeks, and then planted in moist potting soil in small pots enclosed in plastic bags to form humid chambers. Germination was scored weekly for four days. Seedlings with open cotyledons were scored as germinated.

### 4.11 Analysis of infructescence size and accelerated decomposition of infructescences

Infructescences were subjected to decomposition tests based on a method modified from the American Wood Protection Association Standard E10 (AWPA, 2016). In brief, mature infructescences collected December 2015 were dried in a 50°C oven, and weighed to obtain initial dry mass per fruit. Infructescences were hydrated by misting with distilled water and being placed in a humidifying room (30°C, 90% humidity) for 6 days. Infructescences were wrapped in individual foil trays and sterilized by autoclaving for 50 min of sterilization at 121°C. Glass 454 ml bottles were filled halfway with moistened potting mixture and a feeder strip of wood; either western hemlock (*Tsuga heretophylla*) or red alder (*Alnus rubra*) was placed on top of the soil. Prepared bottles loosely were capped and sterilized by autoclaving at 121^o^C for 90 min sterilization. Feeder strips were inoculated with a fungus plug of either the white rot fungus *Trametes versicolor* Isolate R‐105, (onto alder feeder strips) or the brown rot fungus *Gloeophyllum trabeum* Isolate Madison 617 (onto hemlock feeder strips). A non‐inoculated set of bottles served as no fungus negative controls. Bottles were incubated at room temperature for 20 days, until the fungus had covered the feeder strips. Bottles lacking fungal growth across the entire length of the feeder strip or any bottles with visual contamination of other fungi were discarded. The hydrated and sterilized sweetgum infructescences were placed into individual bottles on top of the feeder strips. Bottles were incubated at 28°C. A selection of representative bottles was photographed on weekly intervals using a Canon Rebel XSI digital camera. After 30 days of incubation, the colonized infructescences were removed, weighed, dried, and weighed again. The final dry mass was compared to the initial dry mass to determine the change in biomass over the course of the experiment. The non‐inoculated infructescences served as controls for any change in mass due to sample handling and experimental error. As the fungal and sweetgum infructescences were inseparably interconnected the final weight included the total biomass from both fungus and infructescence.

## 5 ACCESSION NUMBERS

*AG1* (GenbankID_AF103903.1), *AG2* (GenbankID_AR227777).

## CONFLICT OF INTEREST

Corresponding author Strauss has directed a university and industry funded research consortium (TBGRC) based at Oregon State University for more than two decades. Its work is directed at producing solutions to the problems of gene dispersal from genetically engineered and exotic trees.

## AUTHORS' CONTRIBUTIONS

Amy L. Klocko analyzed floral and vegetative morphology, conducted qRT‐PCR, took part in analysis of fungal decomposition, and drafted the manuscript. Amy M. Brunner directed creation of the RNAi construct and created the phylogenetic tree. Cathleen Ma directed transformation and propagation. Elizabeth Etherington, Kori Rosenstiel, and Anna Magnuson managed the field trial and field data collections. Barbara J. Taylor did the micro‐CT tomography. Jed Cappellazzi led in execution of the fungal decomposition studies. Thomas Lockwood helped to execute the transformation and in vitro studies. Nichole Covarrubias and Manzhu Bao worked on construct production, transformation, and initial molecular characterization of transgenic plants. Jeffrey J. Morrell directed the fungal decomposition studies. Steven H. Strauss led in obtaining grant funds for the project, designed and directed the overall study, and took part in manuscript preparation and editing.

## Supporting information

Supplementary Material

## References

[pld3225-bib-0001] AWPA . (2016). Standard method of testing wood preservatives by laboratory soil block cultures. AWPA Book of Standards. Birmingham, Alabama: American Wood Protection Association (AWPA).

[pld3225-bib-0002] Bowman, J. L. , Smyth, D. R. , & Meyerowitz, E. M. (1989). Genes directing flower development in Arabidopsis. The Plant Cell, 1, 37–52. 2535466 10.1105/tpc.1.1.37 PMC159735

[pld3225-bib-0003] Bowman, J. L. , Smyth, D. R. , & Meyerowitz, E. M. (1991). Genetic interactions among floral homeotic genes of Arabidopsis. Development, 112, 1–20. 1685111 10.1242/dev.112.1.1

[pld3225-bib-0004] Bruegge, R. V. , Endres, D. , Hautly, A. , Slane, D. , Storgion, M. , Ward, P. , & Schoenberg, R. (2015). City of kirkwood street tree guide. In: COMMISSION, K. U. F. (ed.). Kirkwood: City of Kirkwood.

[pld3225-bib-0005] Brunner, A. M. , Rottmann, W. H. , Sheppard, L. A. , Krutovskii, K. , Difazio, S. P. , Leonardi, S. , & Strauss, S. H. (2000). Structure and expression of duplicate AGAMOUS orthologues in poplar. Plant Molecular Biology, 44, 619–634. 11198423 10.1023/a:1026550205851

[pld3225-bib-0006] CANADA, P. P. (2015). Brazil approves commercial use of genetically modified Eucalyptus. Retrieved from http://www.pulpandpapercanada.com/forestry/brazil-approves-commercial-use-of-genetically-modified-eucalyptus-1003570998

[pld3225-bib-0007] Chang, S. , Mahon, E. L. , Mackay, H. A. , Rottmann, W. H. , Strauss, S. H. , & Pijut, P. M. (2018). Genetic engineering of trees: Progress and new horizons. Vitro Cellular & Developmental Biology, 54, 341–376. 10.1007/s11627-018-9914-1

[pld3225-bib-0008] Coen, E. S. , & Meyerowitz, E. M. (1991). The War of the Whorls ‐ genetic interactions controlling flower development. Nature, 353, 31–37. 1715520 10.1038/353031a0

[pld3225-bib-0009] Davies, B. , Motte, P. , Keck, E. , Saedler, H. , Sommer, H. , & Schwarz‐Sommer, Z. (1999). PLENA and FARINELLI: Redundancy and regulatory interactions between two Antirrhinum MADS‐box factors controlling flower development. The EMBO Journal, 18, 4023–4034. 10.1093/emboj/18.14.4023 10406807 PMC1171478

[pld3225-bib-0010] DAY, A. T. A. 017 . Retrieved from http://www.atreeaday.com/atreeaday/Liquidambar_styraciflua_Worplesdon.html [Online]. [Accessed 12/8/2017].

[pld3225-bib-0011] Dehnen‐Schmutz, K. (2011). Determining non‐invasiveness in ornamental plants to build green lists. Journal of Applied Ecology, 48, 1374–1380. 10.1111/j.1365-2664.2011.02061.x

[pld3225-bib-0012] Diaz‐Riquelme, J. , Lijavetzky, D. , Martinez‐Zapater, J. M. , & Carmona, M. J. (2009). Genome‐wide analysis of MIKCC‐type MADS box genes in grapevine. Plant Physiology, 149, 354–369. 10.1104/pp.108.131052 18997115 PMC2613716

[pld3225-bib-0013] Dong, Y. , & Wang, Y. Z. (2015). Seed shattering: From models to crops. Frontiers in Plant Science, 6, 476 10.3389/fpls.2015.00476 26157453 PMC4478375

[pld3225-bib-0014] Dreni, L. , & Kater, M. M. (2014). MADS reloaded: Evolution of the AGAMOUS subfamily genes. The New Phytologist, 201, 717–732. 24164649 10.1111/nph.12555

[pld3225-bib-0015] Dubois, A. , Raymond, O. , Maene, M. , Baudino, S. , Langlade, N. B. , Boltz, V. , … Bendahmane, M. (2010). Tinkering with the C‐function: A molecular frame for the selection of double flowers in cultivated roses. PLoS ONE, 5, e9288 10.1371/journal.pone.0009288 20174587 PMC2823793

[pld3225-bib-0016] Edgar, R. C. (2004). MUSCLE: A multiple sequence alignment method with reduced time and space complexity. BMC Bioinformatics, 5, 1–19. 15318951 10.1186/1471-2105-5-113 PMC517706

[pld3225-bib-0017] Elorriaga, E. , Meilan, R. , Ma, C. , Skinner, J. S. , Etherington, E. , Brunner, A. , & Strauss, S. H. (2014). A tapetal ablation transgene induces stable male sterility and slows field growth in *Populus* . Tree Genetics & Genomes, 10, 1583–1593. 10.1007/s11295-014-0781-6

[pld3225-bib-0018] Ferrandiz, C. , & Fourquin, C. (2014). Role of the FUL‐SHP network in the evolution of fruit morphology and function. Journal of Experimental Botany, 65, 4505–4513. 10.1093/jxb/ert479 24482369

[pld3225-bib-0019] Fourquin, C. , & Ferrandiz, C. (2012). Functional analyses of AGAMOUS family members in Nicotiana benthamiana clarify the evolution of early and late roles of C‐function genes in eudicots. The Plant Journal, 71, 990–1001. 10.1111/j.1365-313x.2012.05046.x 22563981

[pld3225-bib-0020] Galimba, K. D. , Tolkin, T. R. , Sullivan, A. M. , Melzer, R. , Theissen, G. , & di Stilio, V. S. (2012). Loss of deeply conserved C‐class floral homeotic gene function and C‐ and E‐class protein interaction in a double‐flowered ranunculid mutant. Proceedings of the National Academy of Sciences of the United States of America, 109, E2267–E2275. 10.1073/pnas.1203686109 22853954 PMC3427126

[pld3225-bib-0021] Gambino, G. , Perrone, I. , & Gribaudo, I. (2008). A rapid and effective method for RNA extraction from different tissues of grapevine and other woody plants. Phytochemical Analysis, 19, 520–525. 10.1002/pca.1078 18618437

[pld3225-bib-0022] Garceau, D. C. , Batson, M. K. , & Pan, I. L. (2017). Variations on a theme in fruit development: The PLE lineage of MADS‐box genes in tomato (TAGL1) and other species. Planta, 246, 313–321. 10.1007/s00425-017-2725-5 28660293

[pld3225-bib-0023] Gimenez, E. , Castaneda, L. , Pineda, B. , Pan, I. L. , Moreno, V. , Angosto, T. , & Lozano, R. (2016). Tomato Agamous1 and Arlequin/tomato Agamous‐Like1 MADS‐box genes have redundant and divergent functions required for tomato reproductive development. Plant Molecular Biology, 91, 513–531. 10.1007/s11103-016-0485-4 27125648

[pld3225-bib-0024] Gonsalves, C. , Lee, D. R. , & Gonsalves, D. (2004). Transgenic Virus‐Resistant Papaya: The Hawaiian 'Rainbow' was Rapidly Adopted by Farmers and is of Major Importance in Hawaii Today.

[pld3225-bib-0025] Group, T. A. P. , Chase, M. W. , Christenhusz, M. J. M. , Fay, M. F. , Byng, J. W. , Judd, W. S. , … Stevens, P. F. (2016). An update of the angiosperm phylogeny group classification for the orders and families of flowering plants: APG IV. Botanical Journal of the Linnean Society, 181, 1–20. 10.1111/boj.12385

[pld3225-bib-0026] Hu, J. , Wang, L. , Yan, D. , & Lu, M. Z. (2014). Research and application of transgenic poplar in China InFenning T. (Ed.), Challenges and opportunities for the World's Forests in the 21st Century (pp. 567–584). Netherlands: Springer.

[pld3225-bib-0027] Ickert‐Bond, S. M. , Pigg, K. B. , & Wen, J. (2005). Comparative infructescence morphology in Liquidambar (Altingiaceae) and its evolutionary significance. American Journal of Botany, 92, 1234–1255. 10.3732/ajb.92.8.1234 21646145

[pld3225-bib-0028] Irish, V. (2017). The ABC model of floral development. Current Biology, 27, R887–R890. 10.1016/j.cub.2017.03.045 28898659

[pld3225-bib-0029] Ito, T. , Wellmer, F. , Yu, H. , Das, P. , Ito, N. , Alves‐Ferreira, M. , … Meyerowitz, E. M. (2004). The homeotic protein AGAMOUS controls microsporogenesis by regulation of SPOROCYTELESS. Nature, 430, 356–360. 15254538 10.1038/nature02733

[pld3225-bib-0030] Levia Jr., D. F. , Bollinger III, W. C. , Hrabik Jr., R. A. & Pogge, J. T. (2004). Water storage capacity of empty fruiting heads of *Liquidambar styraciflua* L. (sweetgum)/Capacité de stockage d’eau des bourgeons fruitiers vides de *Liquidambar styraciflua* L. (le copalme d’Amérique). Hydrological Sciences Journal, 49, 843–853. 10.1623/hysj.49.5.843.55133

[pld3225-bib-0031] Kapoor, M. , Tsuda, S. , Tanaka, Y. , Mayama, T. , Okuyama, Y. , Tsuchimoto, S. , & Takatsuji, H. (2002). Role of petunia pMADS3 in determination of floral organ and meristem identity, as revealed by its loss of function. Plant Journal, 32, 115–127. 12366805 10.1046/j.1365-313x.2002.01402.x

[pld3225-bib-0032] Klocko, A. L. , Borejsza‐Wysocka, E. , Brunner, A. M. , Shevchenko, O. , Aldwinckle, H. , & Strauss, S. H. (2016). Transgenic suppression of AGAMOUS genes in apple reduces fertility and increases floral attractiveness. PLoS ONE, 11, e0159421 10.1371/journal.pone.0159421 27500731 PMC4976969

[pld3225-bib-0033] Klocko, A. L. , Brunner, A. M. , Huang, J. , Meilan, R. , Lu, H. , Ma, C. , … Strauss, S. H. (2016). Containment of transgenic trees by suppression of LEAFY. Nature Biotechnology, 34, 918–922. 10.1038/nbt.3636 27606454

[pld3225-bib-0034] Kramer, E. M. , Jaramillo, M. A. , & di Stilio, V. S. (2004). Functional evolution during the diversification of the AGAMOUS subfamily of MADS box genes in angiosperms. Genetics, 166, 1011–1023. 15020484 10.1093/genetics/166.2.1011 PMC1470751

[pld3225-bib-0035] Liljegren, S. J. , Ditta, G. S. , Eshed, Y. , Savidge, B. , Bowman, J. L. , & Yanofsky, M. F. (2000). SHATTERPROOF MADS‐box genes control seed dispersal in Arabidopsis. Nature, 404, 766–770. 10.1038/35008089 10783890

[pld3225-bib-0036] Liu, J. Y. , Huang, Y. H. , Ding, B. , & Tauer, C. G. (1999). cDNA cloning and expression of a sweetgum gene that shows homology with Arabidopsis AGAMOUS. Plant Science, 142, 73–82. 10.1016/s0168-9452(98)00248-9

[pld3225-bib-0037] Liu, Z. , Zhang, D. , Liu, D. , Li, F. , & Lu, H. (2013). Exon skipping of AGAMOUS homolog PrseAG in developing double flowers of Prunus lannesiana (Rosaceae). Plant Cell Reports, 32, 227–237. 10.1007/s00299-012-1357-2 23096754

[pld3225-bib-0038] Lowe, K. , Wu, E. , Wang, N. , Hoerster, G. , Hastings, C. , Cho, M. J. , … Gordon‐Kamm, W. (2016). Morphogenic regulators baby boom and wuschel improve monocot transformation. The Plant Cell, 28, 1998–2015. 10.1105/tpc.16.00124 27600536 PMC5059793

[pld3225-bib-0039] Lu, H. , Klocko, A. L. , Brunner, A. M. , Ma, C. , Magnuson, A. C. , Howe, G. T. , … Strauss, S. H. (2019). RNA interference suppression of AGAMOUS and SEEDSTICK alters floral organ identity and impairs floral organ determinacy, ovule differentiation, and seed‐hair development in Populus. New Phytologist, 222, 923–937. 10.1111/nph.15648 30565259 PMC6590139

[pld3225-bib-0040] Mizukami, Y. , & Ma, H. (1995). Separation of AG function in floral meristem determinacy from that in reproductive organ identity by expressing antisense AG RNA. Plant Molecular Biology, 28, 767–784. 10.1007/bf00042064 7640351

[pld3225-bib-0041] Nagle, M. , Dejardin, A. , Pilate, G. , & Strauss, S. H. (2018). Opportunities for innovation in genetic transformation of forest trees. Frontiers in Plant Science, 9, 1443 10.3389/fpls.2018.01443 30333845 PMC6176273

[pld3225-bib-0042] Ranney, T. G. (2004). Population control: developing non‐invasive nursery crops©. Combined Proceedings International Plant Propagators’ Society, 54, 604–607.

[pld3225-bib-0043] Rottmann, W. H. (1999). Liquidambar styraciflua AGAMOUS (LSAG) gene USA patent application.

[pld3225-bib-0044] Sablowski, R. (2007). Flowering and determinacy in Arabidopsis. Journal of Experimental Botany, 58, 899–907. 10.1093/jxb/erm002 17293602

[pld3225-bib-0045] Scorza, R. , Hily, J.‐M. , Callahan, A. , Malinowski, T. , Cambra, M. , Capote, N. , … Ravelonandro, M. (2007). Deregulation of plum pox resistant transgenic plum "honeysweet". Acta Horticulturae, 738, 669–673. 10.1007/978-94-007-2156-2_12

[pld3225-bib-0046] Senthil‐Kumar, M. , & Mysore, K. S. (2011). Caveat of RNAi in plants: The off‐target effect. Methods in Molecular Biology, 744, 13–25. 21533683 10.1007/978-1-61779-123-9_2

[pld3225-bib-0047] Soltis, D. E. , Mort, M. E. , Latvis, M. , Mavrodiev, E. V. , O'Meara, B. C. , Soltis, P. S. , … Rubio De Casas, R. (2013). Phylogenetic relationships and character evolution analysis of Saxifragales using a supermatrix approach. American Journal of Botany, 100, 916–929. 10.3732/ajb.1300044 23629845

[pld3225-bib-0048] Staton, M. , Best, T. , Khodwekar, S. , Owusu, S. , Xu, T. , Xu, Y. , … Carlson, J. E. (2015). Preliminary genomic characterization of ten hardwood tree species from multiplexed low coverage whole genome sequencing. PLoS ONE, 10, e0145031 10.1371/journal.pone.0145031 26698853 PMC4689444

[pld3225-bib-0049] Strauss, S. H. , Jones, K. N. , Lu, H. , Petit, J. D. , Klocko, A. L. , Betts Jr., M. G. , … Needham, M. D. (2017). Reproductive modification in forest plantations: Impacts on biodiversity and society. The New Phytologist, 213, 1000–1021. 10.1111/nph.14374 28079940

[pld3225-bib-0050] Sun, Y. , Fan, Z. , Li, X. , Liu, Z. , Li, J. , & Yin, H. (2014). Distinct double flower varieties in *Camellia japonica* exhibit both expansion and contraction of C‐class gene expression. BMC Plant Biology, 14, 288. 25344122 10.1186/s12870-014-0288-1 PMC4219040

[pld3225-bib-0051] Swearingen, J. , & Bargeron, C. (2016). Invasive Plant Atlas of the United States. University of Georgia Center for Invasive Species and Ecosystem Health.

[pld3225-bib-0052] Tamura, K. , Stecher, G. , Peterson, D. , Filipski, A. , & Kumar, S. (2013). MEGA6: Molecular evolutionary genetics analysis version 6.0. Molecular Biology and Evolution, 30, 2725–2729. 10.1093/molbev/mst197 24132122 PMC3840312

[pld3225-bib-0053] Vining, K. J. , Contreras, R. N. , Ranik, M. , & Strauss, S. H. (2012). Genetic methods for mitigating invasiveness of woody ornamental plants: Research needs and opportunities. HortScience, 47, 1210–1216. 10.21273/HORTSCI.47.9.1210

[pld3225-bib-0054] Waltz, E. (2015). Nonbrowning GM apple cleared for market. Nature Biotechnology, 33, 326–327. 10.1038/nbt0415-326c 25850045

[pld3225-bib-0055] Wesley, S. V. , Helliwell, C. A. , Smith, N. A. , Wang, M. B. , Rouse, D. T. , Liu, Q. , … Waterhouse, P. M. (2001). Construct design for efficient, effective and high‐throughput gene silencing in plants. Plant Journal, 27, 581–590. 11576441 10.1046/j.1365-313x.2001.01105.x

[pld3225-bib-0056] Wisniewski, M. , & Bogle, A. L. (1982). The ontogeny of the inflorescence and flower of *Liquidambar‐styraciflua* L (Hamamelidaceae). American Journal of Botany, 69, 1612–1624.

[pld3225-bib-0057] Yamaguchi, T. , & Hirano, H. Y. (2006). Function and diversification of MADS‐box genes in rice. The Scientific World Journal, 6, 1923–1932. 10.1100/tswde.2006.165 17205197 PMC5917342

[pld3225-bib-0058] Yanofsky, M. F. , Ma, H. , Bowman, J. L. , Drews, G. N. , Feldmann, K. A. , & Meyerowitz, E. M. (1990). The protein encoded by the Arabidopsis homeotic gene agamous resembles transcription factors. Nature, 346, 35–39. 10.1038/346035a0 1973265

[pld3225-bib-0059] Zeng, L. , Zhang, N. , Zhang, Q. , Endress, P. K. , Huang, J. , & Ma, H. (2017). Resolution of deep eudicot phylogeny and their temporal diversification using nuclear genes from transcriptomic and genomic datasets. New Phytologist, 214, 1338–1354. 10.1111/nph.14503 28294342

[pld3225-bib-0060] Zhang, C. , Norris‐Caneda, K. H. , Rottmann, W. H. , Gulledge, J. E. , Chang, S. , Kwan, B. Y. , … Hinchee, M. A. (2012). Control of pollen‐mediated gene flow in transgenic trees. Plant Physiology, 159, 1319–1334. 22723085 10.1104/pp.112.197228 PMC3425181

[pld3225-bib-0061] Zhang, N. , Wen, J. , & Zimmer, E. A. (2016). Another look at the phylogenetic position of the grape order Vitales: Chloroplast phylogenomics with an expanded sampling of key lineages. Molecular Phylogenetics and Evolution, 101, 216–223. 10.1016/j.ympev.2016.04.034 27138293

