# Supplementary material for: RNAi of AGAMOUS genes in sweetgum alters reproductive organ identity and decreases fruit persistence
Source: Plant Direct. 2020 May 21;4(5):e00225. doi: 10.1002/pld3.225 (PMC7240341; doi:10.1002/pld3.225)
Supplement: Supplementary file 1 — Supplementary Material [file PLD3-4-e00225-s001.pdf]

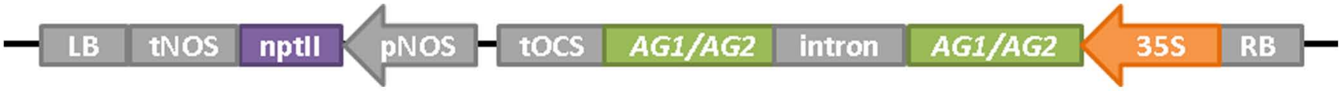

Supplementary Figure 1: Diagram of construct used for sweetgum transformation
A 605 base pair (bp) fragment corresponding to 305 bp of the *Liquidambar styraciflua* gene *AG2* and 300 bp of the gene *AG1* were cloned in sense and antisense orientation in the pART27 vector under control of the constitutive 35S promoter and terminated by the octopine synthase 3' untranslated region (tOCS). This construct contains a neomycin phosphotransferase II (nptII) gene controlled by the constitutive nopaline synthase promoter (pNOS) and terminated by the nopaline synthase 3' untranslated region (tNOS). Arrows indicate the direction of transcriptional activation. The left border and right border are indicated by LB and RB, respectively.

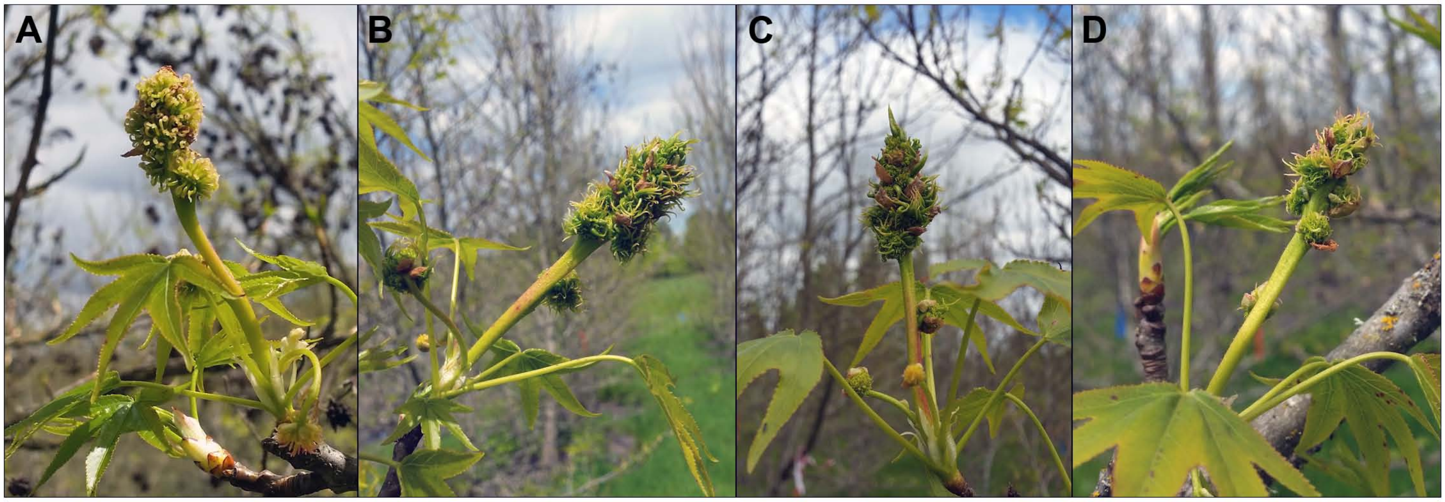

Supplementary Figure 2: Phenotypes were similar in 2017 as previous seasons
Images of female inflorescences from (A) control and RNAi events (B) I7-1, (C) J94-4 and (D) P134-1 captured on April 28, 2017.

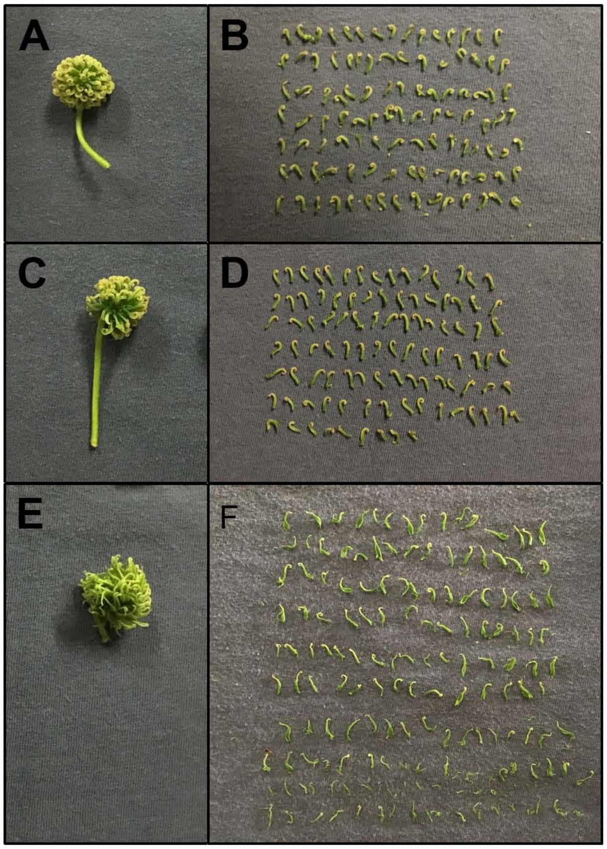

Supplementary Figure 3: Modified RNAi-*AG* inflorescences had diverse organ sizes

Hand dissection of (A, B) control and (C, D) RNAi-*AG* inflorescences with normal structures showed that these inflorescences had styles of regular size, with little variation. (E, F) RNAi-*AG* inflorescences with modified structures showed a diversity of organ sizes. Flowers were collected May of 2018.

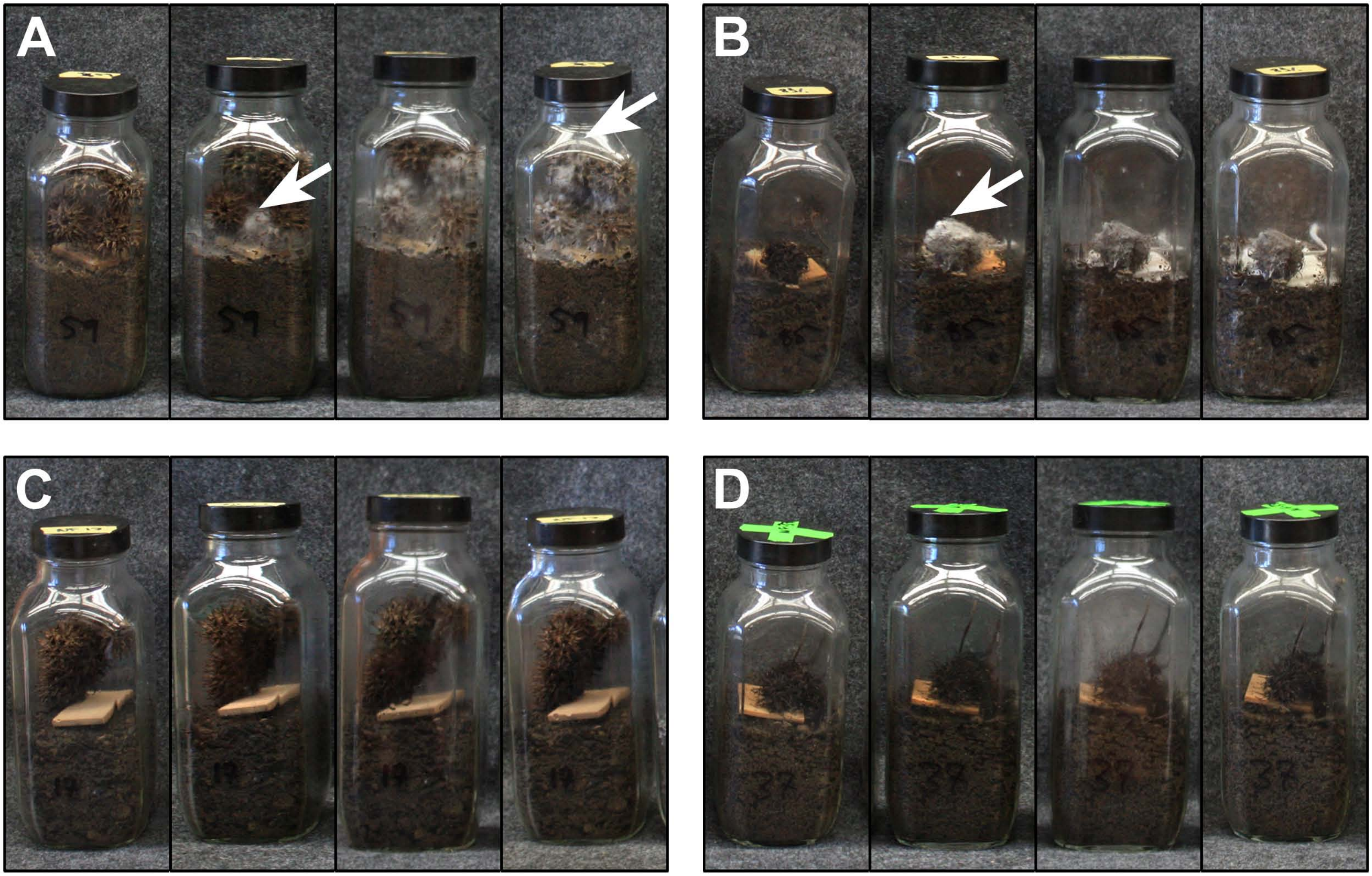

Supplementary Figure 4: Infructescences were colonized well by fungi
Bottles show representative images of (A) wild-type infructescences with white rot, (B) modified RNAi infructescences with white rot, (C) wild-type infructescences without any fungal inoculation, and (D) RNAi infructescences without any fungal inoculation. Bottles show a time series from left to right of incubation time of 1 day, 8 days, 15 days and 22 days. Arrows show when the fungus first started to colonize the infructescences and when it reached the apex of the infructescences. Numbers on bottles are from sample randomization.

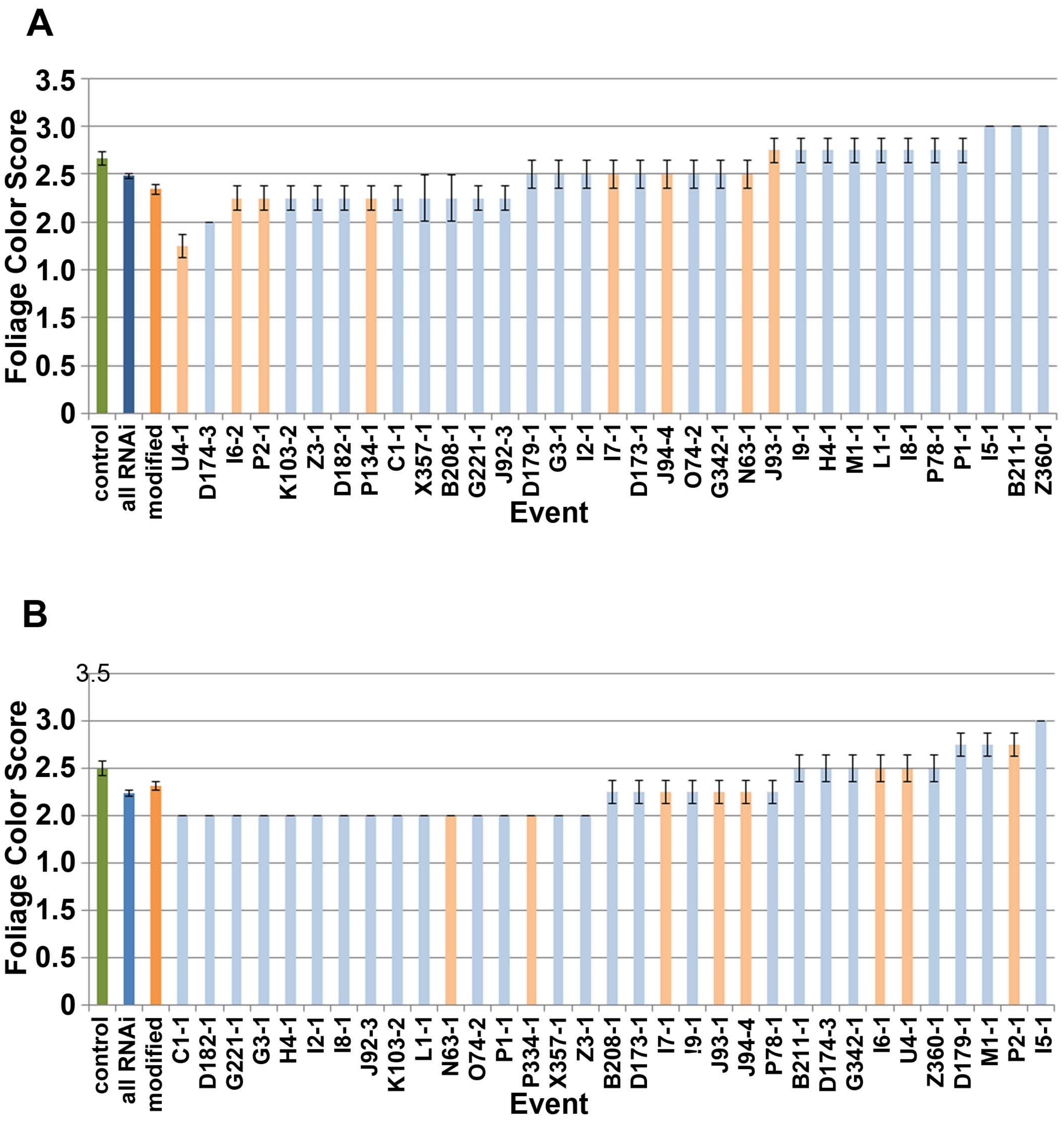

Supplementary Figure 5: Quantification of fall foliage coloration
Foliage coloration was scored on a relative numeric scale. Average fall color scores by event from (A) 2007 and (B) (2012). Dark green bar is the average of control trees, dark blue bar is the average across events of all RNAi-*AG* trees, dark orange bar is the average across events of all RNAi-*AG* trees modified flowers, pale blue bars show events without floral modifications (either non-flowering or normal flowering), pale orange bars show events with modified flowers. Error bars show standard error of trees per event.

Supplementary Table: 1 Tree survival by event in 2017

Of the 132 RNAi-*AG* ramets planted, 131 survived until 2016, representing all 33 of the 33 independent events. Of the 12 non-transgenic control trees planted (Non-Tr-Control), all 12 survived until 2017.

Event	Ramets Planted	Surviving Ramets	% Survival
B208-1	4	4	100.0
B211-1	4	4	100.0
C1-1	4	4	100.0
D173-1	4	4	100.0
D174-3	4	4	100.0
D179-1	4	4	100.0
D182-1	4	4	100.0
G221-1	4	4	100.0
G3-1	4	4	100.0
G342-1	4	4	100.0
H4-1	4	4	100.0
I2-1	4	4	100.0
I5-1	4	3	75.0
I6-2	4	4	100.0
I7-1	4	4	100.0
I8-1	4	4	100.0
I9-1	4	4	100.0
J92-3	4	4	100.0
J93-1	4	4	100.0
J94-4	4	4	100.0
K103-2	4	4	100.0
L1-1	4	4	100.0
M1-1	4	4	100.0
N63-1	4	4	100.0
O74-2	4	4	100.0
P1-1	4	4	100.0
P134-1	4	4	100.0
P2-1	4	4	100.0
P78-1	4	4	100.0
U4-1	4	4	100.0
X357-1	4	4	100.0
Z3-1	4	4	100.0
Z360-1	4	4	100.0
**All RNAi-*AG* ramets**	**132**	131	**99.2**
**All RNAi-*AG* events**	**33**	33	**100.0**
**NonTr-Control**	**12**	12	**100.0**

Supplementary Table 2: Tree flowering by event in 2014

Of the 131 RNAi-*AG* ramets, 10 flowered in 2014, representing 7 of the 33 independent events. Of the 12 non-transgenic control trees (Non-Tr-Control), 2 flowered in 2014. Flowering events are shown in bold and shaded in grey.

Event	Total ramets	Flowering ramets	% flowering
B208-1	4	0	0.0
B211-1	4	0	0.0
C1-1	4	0	0.0
D173-1	4	0	0.0
D174-3	4	0	0.0
D179-1	4	0	0.0
D182-1	4	0	0.0
G221-1	4	0	0.0
G3-1	4	0	0.0
G342-1	4	0	0.0
H4-1	4	0	0.0
I2-1	4	**1**	**25.0**
I5-1	4	0	0.0
I6-2	4	0	0.0
I7-1	4	**1**	**25.0**
I8-1	4	0	0.0
I9-1	4	0	0.0
J92-3	4	0	0.0
J93-1	4	0	0.0
J94-4	4	**2**	**50.0**
K103-2	4	0	0.0
L1-1	4	0	0.0
M1-1	4	0	0.0
N63-1	4	**2**	**50.0**
O74-2	4	0	0.0
P1-1	4	**1**	**25.0**
P134-1	4	**2**	**50.0**
P2-1	4	**1**	**25.0**
P78-1	4	0	0.0
U4-1	4	0	0.0
X357-1	4	0	0.0
Z3-1	4	0	0.0
Z360-1	4	0	0.0
**All RNAi-*AG* ramets**	**131**	**10**	**7.6**
**All RNAi-*AG* events**	**33**	**7**	**21.2**
**NonTr-Control**	**12**	**2**	**16.7**

Supplementary Table 3: Tree flowering by event in 2015

Of the 131 RNAi-*AG* ramets, 31 flowered in 2015, representing 17 of the 33 independent events. Of the 12 non-transgenic control trees (Non-Tr-Control), 4 flowered in 2015. Flowering events are shown in bold and shaded in grey.

Event	Total ramets	Flowering ramets	% flowering
B208-1	4	0	0.0
**B211-1**	**4**	**1**	**25.0**
C1-1	4	0	0.0
**D173-1**	**4**	**3**	**75.0**
D174-3	4	0	0.0
D179-1	4	0	0.0
D182-1	4	0	0.0
G221-1	4	0	0.0
**G3-1**	**4**	**2**	**50.0**
**G342-1**	**4**	**1**	**25.0**
H4-1	4	0	0.0
**I2-1**	**4**	**2**	**50.0**
I5-1	4	0	0.0
**I6-2**	**4**	**1**	**25.0**
**I7-1**	**4**	**2**	**50.0**
**I8-1**	**4**	**1**	**25.0**
I9-1	4	0	0.0
J92-3	4	0	0.0
**J93-1**	**4**	**2**	**50.0**
**J94-4**	**4**	**2**	**50.0**
K103-2	4	0	0.0
L1-1	4	0	0.0
M1-1	4	0	0.0
**N63-1**	**4**	**2**	**50.0**
**O74-2**	**4**	**2**	**50.0**
**P1-1**	**4**	**2**	**50.0**
**P134-1**	**4**	**2**	**50.0**
**P2-1**	**4**	**2**	**50.0**
**P78-1**	**4**	**2**	**50.0**
**U4-1**	**4**	**2**	**50.0**
X357-1	4	0	0.0
Z3-1	4	0	0.0
Z360-1	4	0	0.0
**All RNAi-*AG* ramets**	**131**	31	23.7
**All RNAi-*AG* events**	**33**	17	51.5
**NonTr-Control**	**12**	4	33.3

Supplemental Table 4: Floral Phenotypes by Event in 2014 and 2015

Thirty three independent events represented by 3-4 individual ramets were tested. Flowers were classified as either normal (similar to non-transgenic control trees) or modified (possessing numerous leaf-like projections instead of stigmas). *Event B211-1 had 1 flower that appeared damaged in 2015, +Event D173-1 had 1 flower in 2015 that was too high for quality observation. Flowering events are shaded in grey; events with modified flowers are shown in bold.

Event	Floral Phenotype 2014	Floral Phenotype 2015
B208-1	Non-flowering	Non-flowering
B211-1	Non-flowering	flowering*
C1-1	Non-flowering	Non-flowering
D173-1	Non-flowering	flowering+
D174-3	Non-flowering	Non-flowering
D179-1	Non-flowering	Non-flowering
D182-1	Non-flowering	Non-flowering
G221-1	Non-flowering	Non-flowering
G3-1	Non-flowering	normal
G342-1	Non-flowering	normal
H4-1	Non-flowering	Non-flowering
I2-1	normal	normal
I5-1	Non-flowering	Non-flowering
**I6-2**	Non-flowering	**modified**
**I7-1**	**modified**	**modified**
I8-1	Non-flowering	normal
I9-1	Non-flowering	Non-flowering
J92-3	Non-flowering	Non-flowering
**J93-1**	Non-flowering	**modified**
**J94-4**	**modified**	**modified**
K103-2	Non-flowering	Non-flowering
L1-1	Non-flowering	Non-flowering
M1-1	Non-flowering	Non-flowering
**N63-1**	**modified**	**modified**
O74-2	Non-flowering	normal
P1-1	normal	normal
**P134-1**	**modified**	**modified**
**P2-1**	**modified**	**modified**
P78-1	Non-flowering	normal
**U4-1**	Non-flowering	**modified**
X357-1	Non-flowering	Non-flowering
Z3-1	Non-flowering	Non-flowering
Z360-1	Non-flowering	Non-flowering
NonTr-Control	normal	normal

Supplementary Table 5: Seed formation and seed viability
Seed formation and seed viability was assayed for three years, beginning with infructescences formed in 2014.

Type	Events analyzed	Trees analyzed	Infructescences analyzed	Total seeds	Viable seeds
**2014**					
Control	1	1	1	1	1
RNAi-*AG* normal	1	1	3	0	0
RNAi-*AG* modified	1	1	1	0	0
**2015**					
Control	1	4	32	101	47
RNAi-*AG* normal	3	5	27	6	0
RNAi-*AG* modified	6	10	65	0	0
**2016**					
Control	1	3	11	159	39
RNAi-*AG* normal	3	5	15	13	2
RNAi-*AG* modified	6	4	9	0	0

Name	ID/Accession	Species	Database
ListAG1	AF103903.1	*Liquidambar styraciflua*	NCBI GenBank
ListAG2	AR227777	*Liquidambar styraciflua*	NCBI GenBank
NelnuAG1	XP_010272685.1	*Nelumbo nucifera*	NCBI GenBank
NelnuAG2	XP_010273432.1	*Nelumbo nucifera*	NCBI GenBank
BevulAG	KMT03859.1	*Beta vulgaris*	NCBI GenBank
PMADS3	X72912	*Petunia x hybrida*	NCBI GenBank
FBP11	X81852	*Petunia x hybrida*	NCBI GenBank
FBP7	X81651	*Petunia x hybrid*	NCBI GenBank
FAR	AB516405	*Antirrhinum majus*	NCBI GenBank
PLE	AB516404	*Antirrhinum majus*	NCBI GenBank
TAG1	L26295	*Solanum lycopersicum*	NCBI GenBank
TAG11	AY098736	*Solanum lycopersicum*	NCBI GenBank
ALQ	ADD38992	*Solanum lycopersicum*	NCBI GenBank
MigutSTK	Migut.C01334.1	*Mimulus guttatus*	Phytozome: Mimulus guttatus v2.0
MigutAG	Migut.M00986.1	*Mimulus guttatus*	Phytozome: Mimulus guttatus v2.0
VvAG1	GSVIVT01000802001	*Vitis vinifera*	Phytozome: Vitis vinifera Genoscope.12X
VvAG2	GSVIVT01021303001	*Vitis vinifera*	Phytozome: Vitis vinifera Genoscope.12X
VvAG3	GSVIVT01025945001	*Vitis vinifera*	Phytozome: Vitis vinifera Genoscope.12X
PalaSTK	AQM56559.1	*Paeonia lactiflora*	NCBI GenBank
PalaAG	AQM56546.1	*Paeonia lactiflora*	NCBI GenBank
PasuAG	AGS12611.1	*Paeonia suffruticosa*	NCBI GenBank
KaladpAG1	Kaladp0053s0086.1	*Kalanchoe fedtschenkoi*	Phytozome: Kalanchoe fedtschenkoi v1.1
KaladpAG2	Kaladp0019s0135.1	*Kalanchoe fedtschenkoi*	Phytozome: Kalanchoe fedtschenkoi v1.1
KaladpSTK1	Kaladp0036s0051.1	*Kalanchoe fedtschenkoi*	Phytozome: Kalanchoe fedtschenkoi v1.1
KaladpSTK2	Kaladp0065s0005.1	*Kalanchoe fedtschenkoi*	Phytozome: Kalanchoe fedtschenkoi v1.1
EucgrSTK	Eucgr.F02981.1	*Eucalyptus grandis*	Phytozome: Eucalyptus grandis v2.0
EucgrAG	Eucgr.E02863.1	*Eucalyptus grandis*	Phytozome: Eucalyptus grandis v2.0
EucgrSHP	Eucgr.K01195.1	*Eucalyptus grandis*	Phytozome: Eucalyptus grandis v2.0
PotriAG1	Potri.004G064300.2	*Populus trichocarpa*	Phytozome: Populus trichocarpa v3.0
PotriAG2	Potri.011G075800.1	*Populus trichocarpa*	Phytozome: Populus trichocarpa v3.0
PotriSTK1	Potri.013G104900.1	*Populus trichocarpa*	Phytozome: Populus trichocarpa v3.0
PotriSTK2	Potri.019G077200.1	*Populus trichocarpa*	Phytozome: Populus trichocarpa v3.0
AG	At4g18960	*Arabidopsis thaliana*	Phytozome: Arabidopsis thaliana TAIR10
SHP1	At3g58780	*Arabidopsis thaliana*	Phytozome: Arabidopsis thaliana TAIR10
SHP2	At2g42830	*Arabidopsis thaliana*	Phytozome: Arabidopsis thaliana TAIR10
STK	At4g09960	*Arabidopsis thaliana*	Phytozome: Arabidopsis thaliana TAIR10
PrupeAG	Prupe.4G070500.1	*Prunus persica*	Phytozome: Prunus persica v2.1
PrupeSHP	Prupe.3G170600.1	*Prunus persica*	Phytozome: Prunus persica v2.1
PrupeSTK	Prupe.1G549600.1	*Prunus persica*	Phytozome: Prunus persica v2.1
MedtrSHP	Medtr3g452380.1	*Medicago truncatula*	Phytozome: Medicago truncatula Mt4.0v1
MedtrAGa	Medtr2g017865.1	*Medicago truncatula*	Phytozome: Medicago truncatula Mt4.0v1
MedtrAGb	Medtr8g087860.1	*Medicago truncatula*	Phytozome: Medicago truncatula Mt4.0v1
CejaAG1	ASY97759.1	*Cercidiphyllum japonicum*	NCBI GenBank
CejaAG2	ASY97760.1	*Cercidiphyllum japonicum*	NCBI GenBank
CejaAG11	ASY97762.1	*Cercidiphyllum japonicum*	NCBI GenBank
AqcoeAG1	Aqcoe4G024000.1	*Aquilegia coerulea*	Phytozyome: Aquilegia coerulea v3.1
AqcoeAG2	Aqcoe4G024100.1	*Aquilegia coerulea*	Phytozyome: Aquilegia coerulea v3.1
AqcoeAG3	Aqcoe1G209900.1	*Aquilegia coerulea*	Phytozyome: Aquilegia coerulea v3.1
MediAG	AAS45686.1	*Meliosma dilleniifolia*	NCBI GenBank
PlatAG	AFY98824.1	*Platanus x hispanica*	NCBI GenBank

Supplemental Table 6: Gene names, ID/Accessions and species used for analysis of sweetgum *AG*-like genes

Predicted full-length proteins from a variety of eudicot species were selected for creation of an *AG* phylogenic tree.

Specific methods for creating 3D reconstructions of individual sweetgum flowers in the Avizo 9.4 program:

1) From the original complete data set, slices that included flowers associated with one genotype sample were collected into a subvolume.
2) From this first subvolume, a smaller subvolume was created to isolate all of the slices for a single flower from many of the segments of other flowers and to reduce the size of the cylinder for processing. The flower chosen for further processing was denoted by its location within the genotype subvolume, such as left or upper flower.
3) Next steps in order:
   a) The smaller data set subvolume was processed with the Deblur function using a sharpening factor of 4. This process helped to remove blurring due to the spiral trajectory of the X-rays during scanning. This subvolume was also cropped in order to eliminate some pixels of the cylinder wall and other flowers prior to subsequent processing of the selected flower.
   b) Further filtering was done using the Median Filter module (3D, iteration, at 26 pixels, which smoothed noise in the image but preserved edges. This filtered image was saved for later processing steps.
   c) Processing with the Interactive Thresholding module was used to separate the image of the selected flower from that of the sheets of parafilm, which separated flowers of different genotypes, and adjacent flowers. The parafilm image had similar pixel intensities to flowers.
      i) By manually adjusting the threshold setting, pixel intensities for the flower image (as the mask color) could be selected that largely excluded pixels of the parafilm image and the cylinder wall. Although this process worked well where the flower and parafilm or cylinder wall were physically separate, pixels in slices where the flower was in contact with the parafilm or wall usually were not separable and included as part of the flower to be manually separated in later processing steps.
      ii) This image produced by this coarse filtering step was used to generate a mask needed for later steps. After this step of the processing, the pixels selected for the flower image were often smaller than of the actual flower image. This discrepancy was corrected later but was needed in order to separate the parafilm and flower image elements.
   d) After a round of Interactive Thresholding, the data set was processed using Connected Components to separate other flower sections and parafilm regions from the flower of interest. In this module, pixels belonging to the element will have the same color and the connection size was set at 100,000 pixels minimum. For some flowers, there were many areas of overlap between the parafilm and neighboring flowers requiring additional processing to separate them.
   e) To isolate the flower chosen for 3D surface rendering, it was necessary to move to the Thresholding tab and load the filtered data cylinder for further processing.
      i) Each pixel color for a connected component is loaded into this tab. The main flower pixel color is highlighted and displayed in the X-Y, X-Z and Y-Z planes.
      ii) Selecting the pointer and the cut icon, the entire data set was examined to identify regions in which the flower pixels are connected to other flowers or parafilm and the

pixels forming the linkage were cut away manually. An iterative process in the three dimensions was necessary in order to discover and dissect all of the false linkages.

iii) After a round of cutting in which the changes transfer to the file in the process tab, the data set was run through the Connected Component module to confirm separation of the different elements. In some regions, linkages to the parafilm were permitted at this stage and removed later in the final surface image through a cropping and editing process.

iv) At the end of this process, the pixels of the selected flower have a single color identity.

f) With the final separated flower, processing in the Interactive Threshold module was used to make the pixels in the selected flower have a single intensity; this is a binary image data set of a single flower. This step eliminates visualization of all of the other flower elements and parafilm.

g) To restore the pixels to the selected flower that were removed to facilitate separation of extraneous elements, an enlarged version of the flower is created. Pixels were added back to the flower data set in the Dilation module (type= cube; interpretation= 3D; neighbor =26 [higher resolution]; size =3 or 5 pixels). The number of pixels was chosen to be 3 or 5 depending on which setting restored most of the pixels lost to the thresholding step. The enlarged version is used as a mask for the next segmentation step.

h) In the Mask module, the input file is the original de-blurred and filtered file. The binary input image is the Dilated file. This file is one in which only the pixels of the isolated flower are visible and pixels around the flower sections form the mask. This means that the flower image is included along with surrounding pixels of the mask, which can now be processed through segmentation modules.

i) In the Thresholding tab, the masked original file is loaded as the data. By selecting the threshold for voxels of the flower image away from the surrounding voxels of the mask and then using Watershed segmentation to generate a new image of the flower.

j) In the Process tab, the segmented flower file (which was renamed) was highlighted and the Generating Surface module was chosen. The default settings were used and the file reduced in size by the Compactify module. In some cases, there were remaining parafilm or processes of adjacent flowers which were removed using the surface editor tool. The final surface file is saved.

k) Images of the three-dimensional surface view rotated in different planes were captured by the Snapshot tool in the header and then saved as TIF files.
